# Exploring the Role of RhoA/ROCK Signaling in Pain: A Narrative Review

**DOI:** 10.14336/AD.2024.1539

**Published:** 2025-03-13

**Authors:** Nan Chen, Ye Tu, Dai-Qiang Liu, Yi Zhang, Yu-Ke Tian, Ya-Qun Zhou, Shao-Bing Yang

**Affiliations:** Department of Anesthesiology and Pain Medicine, Hubei Key Laboratory of Geriatric Anesthesia and Perioperative Brain Health, and Wuhan Clinical Research Center for Geriatric Anesthesia, Tongji Hospital, Tongji Medical College, Huazhong University of Science and Technology, Wuhan, 430030, China

**Keywords:** Pain, RhoA, ROCK, Ion channel, Neuroinflammation, Synaptic plasticity

## Abstract

Despite significant progress in understanding the mechanisms of pain and developing therapeutic agents, pain remains a challenging and unresolved clinical issue. The Ras homolog gene family member A (RhoA), a member of the small guanosine triphosphate hydrolases (GTPases) of the Ras homolog family, is involved in transmitting signals that regulate various cellular processes. RhoA exerts its effects through a range of downstream effectors, with Rho-associated kinase (ROCK) being the most extensively studied. Emerging evidence suggests that the RhoA/ROCK signaling pathway plays a crucial role in pain transmission and sensitization. Our work indicates that targeting the RhoA/ROCK signaling pathway may offer a promising therapeutic avenue for alleviating pain.

## 1. Introduction

Pain is regarded as a global public health concern, severely impacting patient’s quality of life [1]. Despite significant advancements in understanding the mechanisms of pain and the development of therapeutic drugs, it remains a challenging and unresolved clinical issue [2, 3]. Current analgesics exhibit limited efficacy or are often restricted in clinical use due to undesirable side effects. Thus, a comprehensive understanding of the mechanisms underlying pain is essential for developing effective analgesics.

Ras homolog (Rho)-family small guanosine triphosphate hydrolases (GTPases) are key regulators of cell adhesion and the dynamics of the cytoskeleton [4]. Among the 20 members of the Rho-family small GTPases in humans, Ras homolog gene family member A (RhoA) is one of the most extensively studied [5]. Rho-associated coiled-coil-containing protein kinase (Rho kinase; ROCK), a serine/threonine protein kinase, is the primary downstream effector of RhoA [6]. The RhoA/ROCK signaling pathway plays a vital role in regulating numerous cellular processes, including cytoskeletal rearrangement, morphogenesis, phagocytosis, migration, proliferation, intracellular transport, and plasticity [7, 8]. Abnormal activation of the RhoA/ROCK pathway has been implicated in several central nervous system (CNS) disorders, such as stroke, Alzheimer’s disease, and traumatic brain injury [9, 10]. Importantly, increasing evidence highlights the significant role of the RhoA/ROCK pathway in pain. This review aims to elucidate the molecular mechanisms by which dysregulated RhoA/ROCK signaling contributes to pain, emphasizing the critical role of this pathway in pain modulation and the potential therapeutic benefits of pharmacological inhibitors targeting it.

## 2. An overview of the RhoA/ROCK signaling pathway

Similar to most small GTPases, RhoA functions as a molecular switch, alternating between an inactive guanosine diphosphate (GDP)-bound state and an active guanosine triphosphate (GTP)-bound state [11]. This conformational shift is mediated by various regulatory proteins through the processes of GDP/GTP exchange and GTP hydrolysis. Guanine nucleotide exchange factors (GEFs) activate RhoA by facilitating the release of GDP and the binding of GTP [12]. In contrast, GTPase-activating proteins (GAPs) promote the hydrolysis of GTP into GDP and inorganic phosphate (Pi), leading to the inactivation of RhoA. Guanine nucleotide dissociation inhibitors (GDIs) prevent RhoA activation by obstructing the dissociation of GDP from RhoA in the cytosol. Furthermore, before activation by GEFs, RhoA must dissociate from GDI, as GEFs cannot directly interact with the GTPase-GDI complex. GDI displacement factors (GDFs) facilitate this dissociation, allowing RhoA to be activated by GEFs. A prior review has provided a comprehensive summary of GEFs, GAPs, GDIs, and GDFs that target RhoA [13]. Additionally, post-translational modifications (PTMs) such as prenylation, phosphorylation, and ubiquitination also influence the subcellular localization and activity of RhoA [13]. Furthermore, the expression of RhoA can be modulated by several transcription factors and is subject to post-transcriptional regulation by microRNAs (miRNAs) [14]. Details regarding the specific types of PTMs, as well as the transcriptional and post-transcriptional regulation of RhoA, can be found in the previous review [13].

Various extracellular signals can trigger the activation of RhoA via interactions with membrane receptors. Once activated, GTP-bound RhoA transmits signals to downstream effectors, with ROCK being one of the most well-characterized. There are two isoforms of ROCK: ROCK1 and ROCK2. These isoforms display a high degree of homology, sharing 65 % of amino acid sequence and 92 % of kinase domain. ROCK1 is primarily found in the lungs, liver, and other non-neuronal tissues, while ROCK2 is predominantly expressed in the brain, spinal cord, and muscles [15]. Notably, a growing body of evidence implicates the RhoA/ROCK pathway in the regulation of nociceptive transmission and central sensitization, highlighting its significant role in pain.

## 3. Aberrant activation of RhoA/ROCK signaling pathways in pain

A growing number of studies have demonstrated the dysregulated activation of the RhoA/ROCK signaling pathways in the dorsal root ganglia (DRG) and spinal cord in rodent pain models. Preclinical studies have shown that spinal ROCK2 expression or ROCK2 immunoreactivity is elevated in various neuropathic pain models, including partial sciatic nerve injury (SNI) [16], spinal nerve ligation (SNL) [17], tumor cell implantation (TCI) [18], and inflammatory pain induced by formalin [19] or lipopolysaccharide (LPS) [20]. Furthermore, a rise in phosphorylated ROCK2 (p-ROCK2), rather than total ROCK2 levels, has been documented in the spinal cords of rats subjected to TCI and mice undergoing chronic constriction injury (CCI) of the sciatic nerves [21, 22]. Interestingly, studies have shown that ROCK1 protein levels were elevated in the DRG and spinal cords of CCI rats, while ROCK2 levels remained stable [23, 24]. Similarly, increased ROCK1 expression has also been observed in the spinal cords of rats with methylmercury (MeHg)-induced neuropathic pain [25]. GTP-bound RhoA and phosphorylated RhoA, along with increased membrane localization of RhoA, represent active RhoA. After intraplantar injection of LPS and formalin, animals demonstrated elevated levels of GTP-bound RhoA and membrane-localized RhoA in the spinal cord [20, 26]. Similarly, SNL led to increased membrane localization of RhoA, GTP-bound RhoA, and phosphorylated RhoA in the ipsilateral spinal cord [16, 17, 27]. Elevated GTP-bound and membrane-bound RhoA levels were also identified in contused spinal cord tissue following spinal cord injury (SCI) and in the spinal cords of streptozotocin (STZ)-induced diabetic mice [28, 29]. Moreover, increased expression of p-RhoA and RhoA has been reported in the spinal cords of rats with bone cancer pain (BCP) [18, 21]. However, Ke et al. found that carcinoma implantation resulted in the downregulation of spinal RhoA [30]. Additional studies corroborated the increased levels of RhoA, RhoA-GTP, and membrane-localized RhoA in the DRG or spinal cords of rats subjected to CCI [23, 24]. Li et al. also noted heightened expressions of p-RhoA in the spinal cords of CCI mice, while total RhoA levels remained unchanged [22]. In rats with chronic post-thoracotomy pain (CPSP), increased RhoA levels were detected in both the DRG and spinal cord [31]. These findings collectively underscore the aberrant activity of the RhoA/ROCK signaling pathways in pain, suggesting that dysregulated RhoA/ROCK signaling may contribute to pain.

## 4. The role of RhoA/ROCK signaling in pain

Increasing evidence suggests that blocking the RhoA/ROCK signaling pathway pharmacologically can alleviate hyperalgesia and allodynia in animal pain models. In mice, an intraperitoneal injection of the ROCK inhibitor Y27632 elicited an anti-nociceptive response to harmful thermal stimuli [32] and alleviated cold hyperalgesia induced by C7/8 rhizotomy in rats [33]. Extensive studies have shown that intrathecal injections of the C3 exoenzyme (a RhoA inhibitor) or Y27632 significantly diminished pain behaviors in rodent models of neuropathic pain induced by SNL, CCI, TCI, and STZ treatment [16, 17, 29, 30, 34], as well as inflammatory pain induced by LPS and formalin [19, 20, 35]. Inoue et al. demonstrated that pre-administration of C3 exoenzyme and Y-27632 effectively prevented the development of mechanical allodynia and thermal hyperalgesia in mice undergoing partial SNL. However, intrathecal injection of C3 exoenzyme after injury failed to suppress the pain response, underscoring the crucial role of RhoA/ROCK pathway activation in the early stages of neuropathic pain [36]. Mevalonate, a precursor for isoprenoids, facilitates RhoA membrane translocation through isoprenylation [37]. Ohsawa et al. demonstrated that intrathecal delivery of mevalonate promoted RhoA membrane translocation and induced thermal hyperalgesia in naïve mice, which was significantly reduced by prior intrathecal treatment with the geranylgeranyl transferase (GGTase) I inhibitor GGTI-2133 and ROCK inhibitor Y27632 [38]. The results indicate that RhoA activation through geranylgeranylation, driven by mevalonate, contributes to the development of neuropathic pain. Further research has indicated that inhibitors of 3-hydroxy-3-methylglutaryl coenzyme A (HMG-CoA) reductase can suppress mevalonate synthesis, thus impairing the isoprenylation of RhoA. Intrathecal administration of simvastatin, an HMG-CoA reductase inhibitor, reduced formalin-induced inflammatory hyperalgesia, which was partially reversed by mevalonate [38, 39]. Similarly, intraperitoneal or intrathecal administration of simvastatin decreased spinal membrane localization of RhoA and ROCK2 immunoreactivity, alleviating neuropathic pain resulting from partial SNL, CCI, and STZ administration [16, 29, 40]. These findings suggest that the inhibition of RhoA/ROCK signaling by simvastatin reduced the sensitization of spinal nociceptive transmission. The ROCK inhibitor fasudil has also demonstrated potential analgesic effects in certain pain models [41]. When administered intraperitoneally at the highest tested dose of 30 mg/kg, fasudil significantly reduced mechanical allodynia in the models of SNL-induced neuropathic pain, CCI-induced neuropathic pain, CPSP, capsaicin-induced secondary mechanical hypersensitivity, and sodium iodoacetate-induced osteoarthritic pain [31, 41]. It also had modest effects on carrageenan-induced thermal hyperalgesia but did not alleviate mechanical allodynia induced by Complete Freund’s Adjuvant (CFA). Orally administered fasudil demonstrated the capacity to provide rapid, temporary pain relief in rats suffering from monoiodoacetate-induced arthritis (MIA) and adjuvant-induced arthritis (AIA) [42]. Additionally, the intrathecal administration of fasudil mitigated mechanical allodynia and thermal hyperalgesia in rats with TCI [21]. Kishima et al. reported that intraperitoneal administration of the ROCK inhibitor ripasudil reduced SCI-induced mechanical allodynia at 14 and 28 days [43]. Notably, Y27632 and fasudil exhibited dual effects on nociceptive responses when injected intraplantar, with lower doses producing pronociceptive effects and higher doses producing antinociceptive responses in carrageenan-induced inflammatory pain models [44]. Although fasudil and Y27632 demonstrated low specificity for ROCK, H-1152 was identified as a specific, potent, and membrane-permeable ROCK inhibitor, significantly decreasing ipsilateral mechanical pain in spinal nerve-transected mice [45]. Tatsumi et al. demonstrated that early administration of H1152 (24 pmol, 240 pmol, intrathecally) notably delayed the onset of ipsilateral tactile allodynia resulting from spinal nerve injury, whereas delayed administration of H1152 did not reduce mechanical allodynia [46]. AS1892802, a novel and highly selective ROCK inhibitor with limited ability to penetrate the CNS, exhibited robust antinociceptive effects in both AIA and MIA models when administered orally or intra-articularly [42, 47]. Moreover, repeated oral administration of AS1892802 provided a prolonged and potent analgesic effect in STZ-induced diabetic neuropathic pain and MIA rats [48]. Pretreatment with extradural administration of BA-210, a cell-permeable fusion protein derived from C3 transferase, did not prevent allodynia six weeks after spinal cord contusion [49]. In contrast, lumbar injection of siRhoA significantly reduced tactile hypersensitivity at 6 to 8 weeks after contusive SCI [28]. The difference may be due to the prolonged effect of siRNA in decreasing RhoA protein levels. Several studies have shown that paeonol, ibuprofen, and ferulic acid mitigated thermal hyperalgesia in rat models of CCI by inhibiting RhoA/ROCK signaling activation [23, 24]. Additionally, treatment with epigallocatechin-3-gallate reduced thermal hyperalgesia following SCI by down-regulating RhoA expression in mice [50]. Knockdown of ubiquitin-specific peptidase 53 (USP53) obstructed the activation of FK506 binding protein 51 (FKBP51)/RhoA/ROCK signaling in the spinal cord, reducing CCI-induced mechanical hypersensitivity and thermal hyperalgesia [51]. Previous studies have demonstrated that miRNAs play a key role in pain regulation. MiR-488-3p alleviated neuropathic pain by inhibiting ROCK1 in the DRG of CCI rats, with the reduction in neuropathic pain partially reversed by the intrathecal overexpression of the ROCK1 plasmid [52]. Collectively, these findings underscore the significant role of the RhoA/ROCK signaling pathway in the initiation and development of pain. However, more in-depth explorations are needed to fully understand the precise molecular mechanisms and signaling networks mediated by the RhoA/ROCK pathway.

## 5. Peripheral mechanisms of RhoA/ROCK signaling in pain

### 5.1 RhoA/ROCK signaling and ion channels

**Figure 1.**
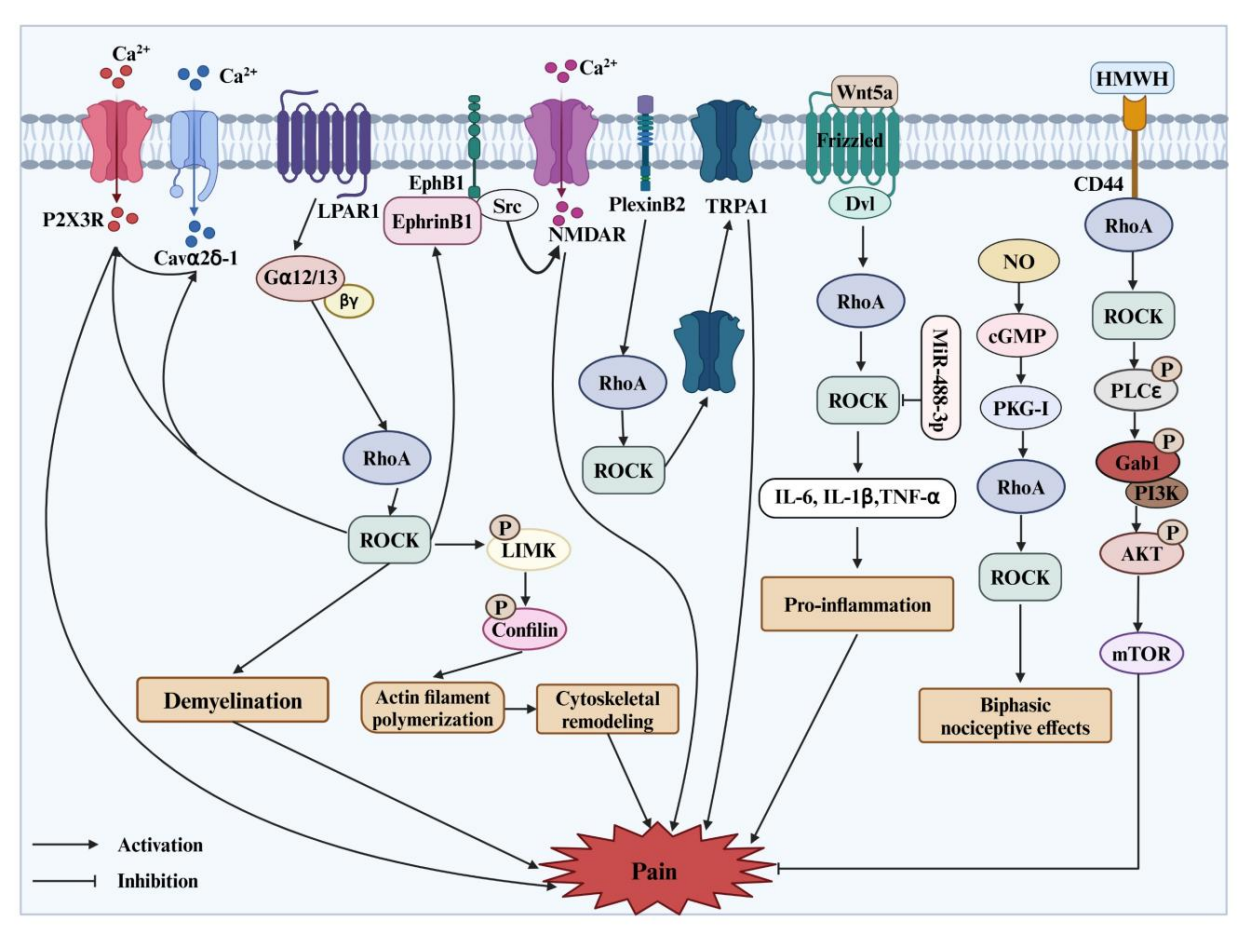
**The peripheral mechanisms of RhoA/ROCK signaling in pain**. RhoA: Ras homolog gene family member A; ROCK: Rho-associated coiled-coil-containing protein kinase; LPAR1: lysophosphatidic acid receptor 1; P2X3R: P2X3 receptor; NMDAR: N-methyl-D-aspartate receptor; TRPA1: transient receptor potential ankyrin 1; LIMK: LIM kinase; Dvl: disheveled; IL-1β: interleukin 1beta; IL-6: interleukin 6; TNF-α: tumor necrosis factor α; NO: nitric oxide; cGMP: cyclic guanosine 3′,5′-monophosphate; PKG-I: protein kinase cGMP-dependent 1; HMWH: High-molecular-weight hyaluronan; CD44: cluster of differentiation 44; PLCε: phospholipase Cε; Gab1: scaffold protein; PI3K: phosphoinositide 3-kinase; AKT: protein kinase B; mTOR: mammalian target of rapamycin. The figure is created in https://www.biorender.com.

The activation of ion channels led to the reduction in threshold or increase in the amplitude of action potentials in DRG neurons, thereby contributing to pain hyperalgesia. Cavα2δ-1, a subunit of voltage-gated calcium channels (VGCCs), facilitates neurotransmitter release, membrane excitability, and synaptic plasticity [53]. Lysophosphatidic acid (LPA), a lipid metabolite released in response to tissue injury, stimulates peripheral nociceptive terminals through the LPA receptor 1 (LPAR1) and its downstream RhoA-ROCK pathway, which has been implicated in the onset of nerve injury-induced neuropathic pain [36]. The upregulation of Cavα2δ-1 in DRG, triggered by LPA (1 nmol, i.t.) and partial SNL, was eliminated following intrathecal pretreatment with C3 exoenzyme and Y-27632. Increased activity of synaptic N-methyl-D-aspartate receptors (NMDARs) is crucial for amplifying nociceptive signals from primary sensory neurons in the context of neuropathic pain. The EphrinB-EphB signaling pathway is associated with the modulation of pain mediated by NMDARs through Src-dependent phosphorylation [54, 55]. Microarray analyses revealed that LPA-induced ephrinB1 expression in the DRG of mice in a RhoA-dependent manner [56]. This study further established that LPAR1/RhoA/ROCK signaling mediates NMDA receptor activation by modulating ephrinB1, thereby initiating behaviors indicative of neuropathic pain following nerve injury. In addition to ephrinB1, four additional genes, including pre-B-cell leukemia transcription factor 3, solute carrier family 8 (sodium/calcium exchanger), member 1, activin A receptor type 1B, and calcium/calmodulin-dependent protein kinase II alpha, being significantly regulated by the LPAR1/RhoA/ROCK signaling pathway, have also been linked to the glutamate-NMDA receptor system [57, 58]. Although further research is warranted to clarify the specific roles of these four genes in LPAR1/RhoA/ROCK signaling-induced neuropathic pain, the NMDAR activation mediated by this signaling cascade stands out as a vital mechanism in peripheral nociception. ATP-gated P2X3 receptor (P2X3R) plays an important role in nociceptive signaling [59]. Wu et al. observed that inhibitors of LPAR1, RhoA, ROCK and P2X3R mitigated bone cancer pain and significantly attenuated the spontaneous responses induced by the P2X3R agonist α, β-meATP [34]. Further research demonstrated that LPA augmented calcium influx prompted by α, β-meATP via P2X3R in primary DRG neurons from rats experiencing bone cancer pain, which could be blocked by the LPAR1 antagonist VPC32183 and the RhoA inhibitor C3 exoenzyme. These results suggested that RhoA functions downstream of LPAR1 in modulating the calcium influx mediated by P2X3R in DRG neurons of BCP rats. Conversely, Qiao et al. demonstrated that LPA enhanced the activity of P2X3R in primary sensory neurons through the activation of LPAR1 and its downstream PKC, rather than the RhoA/ROCK signaling pathway [60]. Further investigation is warranted to determine whether the RhoA/ROCK pathway in the DRG affects the functionality of P2X3R in the context of pain. Additionally, the transient receptor potential ankyrin 1 (TRPA1) ion channel serves as a crucial molecular sensor and mediator of pain signals in DRG neurons, contributing to heightened neuronal excitability [61]. Pharmacological and genetic investigations have highlighted that Semaphorin 4C-Plexin-B2 signaling in peripheral sensory neurons enhances the membrane availability of TRPA1 via the activation of the RhoA/ROCK pathway, ultimately potentiating TRPA1 function and resulting in CFA-induced inflammatory hypersensitivity [62] (Fig 1).

### 5.2 RhoA/ROCK signaling and peripheral pro-inflammatory cytokines

Substantial evidence has highlighted the critical involvement of neuroinflammation in facilitating both peripheral and central sensitization [63]. Research conducted by Fu et al. demonstrated that miR-488-3p mitigated neuropathic pain in a rat model induced by CCI surgery by suppressing the synthesis of pro-inflammatory cytokines (e.g., IL-1β, IL-6, and TNF-α), while simultaneously augmenting the levels of anti-inflammatory factors IL-4 and IL-10 in DRG [52]. The administration of a ROCK1 plasmid via intrathecal injection in these rats led to a partial reversal of the mitigation effects of miR-488-3p on neuropathic pain and neuroinflammation. Another study revealed that the protein levels of Wnt5a, transmembrane receptor Ror2, and the Wnt5a/planar cell polarity pathway member RhoA were increased time-dependently in the thoracic DRG of rats experiencing CPSP [64]. Early inhibition of Wnt5a using Box5 (from postoperative days 0 to 9) significantly reduced mechanical hyperalgesia and reversed RhoA activation, alongside a decrease in IL-1β levels in the DRG. Thus, the activation of the RhoA/ROCK pathway is thought to be linked to the overproduction of pro-inflammatory cytokines, which contributes to the initiation and persistence of neuropathic pain.

### 5.3 RhoA/ROCK signaling and demyelination

Neuropathic pain arising from peripheral and central demyelinating disorders is thought to result from abnormal myelination, indicating that demyelination is a vital mechanism contributing to pain [65]. Intrathecal pretreatment with C3 exoenzyme and Y-27632 effectively eliminated neuropathic pain induced by LPA and nerve injury [36]. Additionally, C3 exoenzyme hindered both LPA- and injury-induced demyelination in the DRG, as demonstrated by stained tissue sections, and prevented the downregulation of myelin-associated proteins, such as myelin basic protein (MBP) and peripheral myelin protein 22 kDa (PMP22). Subsequent studies indicated that demyelination is sustained by the gene silencing of myelin protein gene transcription factor Sox10 and Egr2 through the LPAR1-G12/13-RhoA/ROCK-acetylated NF-κB pathway and the LPAR1-G12/13-RhoA/ROCK-MKK4-JNK-c-Jun pathway respectively [66]. Additional findings implied that the loss of pain transmission through C-fibers is induced by the activation of the LPA1 receptor and its downstream RhoA/ROCK signaling pathway following peripheral nerve injury [67]. The retraction of polymodal C-fiber terminals and the subsequent loss of signaling to dendritic targets in lamina II of the dorsal spinal cord, along with the demyelination of mechanoreceptor Aβ-fibers, followed by the sprouting of Aβ-fibers to establish new connections with the vacated dendrites of second-order nociceptive neurons in lamina II, may underlie the allodynia induced by demyelination [66, 68]. In summary, the activation of the LPAR1/RhoA/ROCK pathway drives demyelination in the DRG, thereby playing a significant role in the development of allodynia and hyperalgesia following nerve injury.

### 5.4 RhoA/ROCK signaling and the actin cytoskeleton remodeling

The RhoA/ROCK pathway is crucial to regulate actin filaments, which are vital components of the cytoskeleton [69]. The study has shown that RhoA can inactivate the actin depolymerizing factor cofilin by phosphorylating LIMK [70]. Inhibition of the RhoA/LIMK/Cofilin pathway in the DRG using Y-27632 and simvastatin considerably alleviated heat hyperalgesia and mechanical allodynia in rats subjected to CCI [40]. Activation of the RhoA/LIMK/cofilin pathway promotes actin filament polymerization, disrupts cytoskeletal organization, and aids in the intracellular transport of various nociceptive signaling molecules. The cytoskeleton serves as a structural framework that supports the transport of nociceptive signaling molecules, highlighting the essential role of RhoA/ROCK in the regulation of pain signal transmission.

### 5.5 Other mechanisms of RhoA/ROCK signaling in peripheral pain processing

Importantly, the RhoA/ROCK signaling pathway has a complex role in regulating peripheral pain perception. Paiva-Lima et al. discovered that the intraplantar administration of Y27632 elicited biphasic nociceptive responses in rats [44]. At a lower dosage of 25 μg per paw, Y27632 induced hyperalgesia in naive rats. In contrast, at a higher dosage of 500 μg, Y27632 effectively impeded the reduction of nociceptive thresholds in rats subjected to carrageenan-induced inflammatory pain. This phenomenon is thought to be associated with the nitric oxide (NO)/cyclic guanosine 3′,5′-monophosphate (cGMP)/protein kinase G (PKG) pathway. Similarly, Zulauf et al. suggested that ROCK, by regulating microfilaments, participates in the NO/cGMP/PKG pathway, which is recognized for its biphasic effects on nociception [71]. High-molecular-weight hyaluronan (HMWH) has been reported to alleviate hyperalgesia caused by chemotherapeutic agents such as oxaliplatin and paclitaxel [72], as well as various inflammatory mediators, including carrageenan, PGE2, epinephrine, TNF-α, and IL-6 [73-75]. HMWH binds to a cluster of differentiation 44 (CD44) on nociceptors, inhibiting the sensitization of cultured small-diameter DRG neurons elicited by PGE2 [73]. Recent studies indicated that the intradermal injection of Y27632 (1 μg) into the hind paw reduced HMWH-induced anti-hyperalgesia in models of PGE2-induced inflammatory pain and neuropathic pain induced by oxaliplatin and paclitaxel [72, 73]. Additionally, Bonet et al. demonstrated that HMWH attenuated inflammatory hyperalgesia through its action on CD44 and the subsequent activation of the downstream RhoA/phospholipase C/phosphoinositide 3-kinase γ/protein kinase B/mammalian target of rapamycin signaling pathway [73, 74]. These findings underscore the importance of the RhoA/ROCK pathway in HMWH-induced anti-hyperalgesia and the reversal of nociceptor sensitization.

## 6. Spinal mechanisms of RhoA/ROCK signaling in pain

### 6.1 RhoA/ROCK signaling and Neuroinflammation

**Figure 2.**
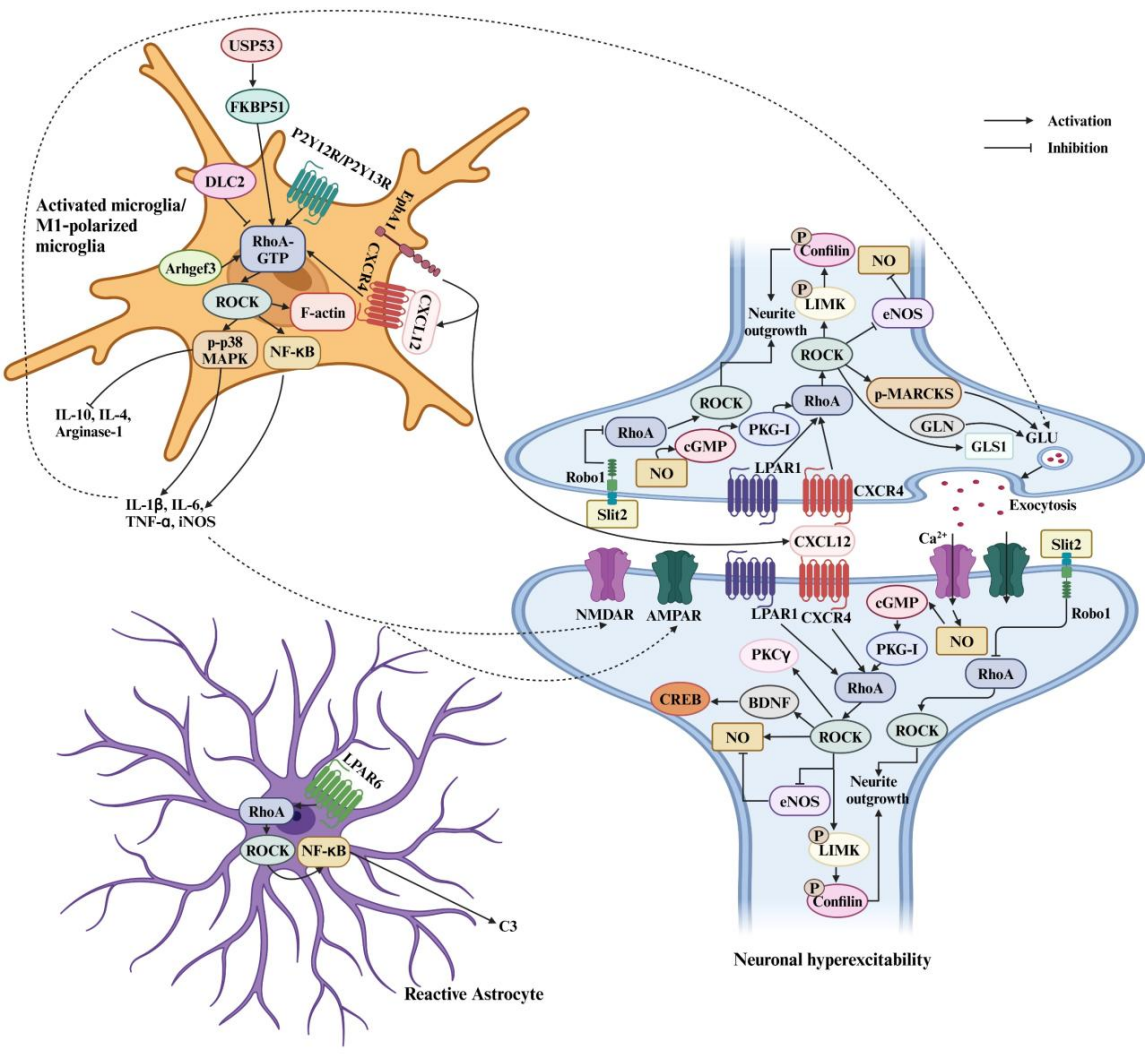
**The spinal cord mechanisms of RhoA/ROCK signaling in pain**. RhoA: Ras homolog gene family member A; ROCK: Rho-associated coiled-coil-containing protein kinase; LPAR1: lysophosphatidic acid receptor 1; CXCR4: C-X-C motif chemokine receptor 4; CXCL12: C-X-C motif chemokine ligand 12; P2Y12R: P2Y12 receptor; DLC2: deficiency of deleted in liver cancer 2; USP53: ubiquitin-specific peptidase 53; FKBP51: FK506 binding protein 51; TRPA1: transient receptor potential ankyrin 1; LIMK: LIM kinase; eNOS: endothelial nitric oxide synthetase; NO: nitric oxide; MAPK: mitogen-activated protein kinases; IL-1β: interleukin 1beta; IL-6: interleukin 6; TNF-α: tumor necrosis factor α; IL-10: interleukin 10; IL-4: interleukin 4; NF-κB: nuclear factor kappa B; CREB: CRE binding protein; BDNF: brain-derived neurotrophic factor; iNOS: inducible nitric oxide synthase; cGMP: cyclic guanosine 3′,5′-monophosphate; PKG-I: protein kinase cGMP-dependent 1; PKCγ: protein kinase Cγ; NMDAR: N-methyl-D-aspartate receptor; AMPAR: α-amino-3-hydroxy-5-methyl-4-isoxazole-propionic acid receptor; MARCKS: myristoylated alanine-rich protein kinase C substrate; GLS1: glutaminase 1; GLN: glutamine; GLU: glutamate. The figure is created in https://www.biorender.com.

A growing body of evidence indicates that spinal neuroinflammation plays a pivotal role in the onset and progression of pain. Neuroinflammation in the CNS is marked by the activation of microglia and astrocytes, excessive production of proinflammatory cytokines and chemokines, and the infiltration of immune cells [76, 77] (Fig. 2).

### 6.1.1 Activation of microglia

Initial evidence indicated that the activation of RhoA contributed to the development of pain following spinal cord contusion, primarily due to the activation or recruitment of macrophages and microglia at the injury site [78]. Subsequently, RhoA/ROCK signaling has been shown to mediate both inflammatory pain and neuropathic pain through the activation of p38 mitogen-activated protein kinase (MAPK) in spinal microglia [26, 43, 46]. Microglial ATP receptors in the spinal cord, including the P2Y metabotropic G-protein-coupled purinergic receptors P2Y12 and P2Y13, are crucial in pain [17, 79]. Intrathecal administration of the P2Y12/P2Y13 receptor agonist 2-(methylthio) adenosine 5'-diphosphate (2Me-SADP) resulted in mechanical allodynia and spinal phosphorylation of p38 MAPK. Inhibition of spinal ROCK with H1152 mitigated mechanical hypersensitivity and prevented both the phosphorylation of spinal p38 MAPK and the upregulation of Iba1 immunoreactivity in 2Me-SADP and nerve-injured rats [17, 46]. Yu et al. also identified a P2Y12 receptor-dependent GTP-RhoA/ROCK2 signaling pathway that mediated the upregulation of p-p38 MAPK and the activation of microglia in the ipsilateral spinal cord of rats subjected to SNL-induced neuropathic pain [17]. These studies highlight that the RhoA/ROCK pathway is responsible for the morphological changes in activated microglia, rather than the number of activated microglia in the dorsal horn. This is because RhoA/ROCK signaling facilitates F-actin stabilization and contributes to the remodeling of the actin cytoskeleton, resulting in changes to cellular morphology. Microglia can be activated into either the pro-inflammatory phenotype (M1) or the anti-inflammatory phenotype (M2). Research has demonstrated that RhoA inhibitor CCG-1423 and p38 MAPK inhibitor SB203580 decreased the proportion of M1-polarized microglia and the expression of pro-inflammatory cytokines (e.g., IL-1β, IL-6, and TNF-α), while simultaneously increasing the proportion of M2-polarized microglia and the levels of anti-inflammatory cytokines (e.g., IL-10) in LPS-treated GMI-R1 microglia [23, 24].

One investigation demonstrated that silencing of Arhgef3, a member of the RhoGEFs that interferes with RhoA activation, inhibited the microglial inflammatory phenotype and decreased the expression of pro-inflammatory cytokines (e.g., IL-1β, IL-6, and TNF-α), and COX-2 in vitro LPS-treated microglia, as well as in an in vivo SCI mouse model [80]. In MeHg-induced neuropathic pain rats, the microglial inflammatory phenotype in the spinal cord was also suppressed by Fasudil through reducing ROCK activity[25]. Deficiency of deleted in liver cancer 2 (DLC2), a Rho GTPase-activating protein, resulted in the inactivation of the RhoA/ROCK pathway. Wong et al. reported that DLC2-knockout mice showed notably higher cumulative pain response times during the inflammatory phase of formalin injection compared to wild-type mice [19]. Additionally, intrathecal injection of C3 exoenzyme or Y27632 meaningly reduced inflammatory pain in DLC2-knockout mice. This treatment also inhibited IL-1β expression, p38 MAPK activation, and decreased the activation of spinal microglia. Taken together, these findings suggest that spinal RhoA/ROCK/p38MAPK signaling contributes to the transformation of microglia into the M1 proinflammatory phenotype, driving the excessive production of cytokines and the development of pain.

### 6.1.2 Activation of astrocytes

Ohsawa et al. proposed that spinal ROCK inhibition with Y27632 or simvastatin reduced the GFAP immune-reactivity in the ipsilateral spinal dorsal horn of SNL mice [16]. This discovery implied that the RhoA/ROCK signaling pathway regulated the reactivity of spinal astrocytes, which is linked to neuropathic pain. In addition, reactive astrocytes can be categorized into two polarization states: the pro-inflammatory phenotype (A1) and the anti-inflammatory phenotype (A2). There is evidence suggesting that the conversion of astrocytes to the A2 phenotype might be of great significance in lessening pain-related behaviors [81]. Y27632 was reported to decrease the activation of spinal A1 astrocytes in SCI rats [82]. In vitro studies showed that Y27632 promoted the transformation of astrocytes into A2 reactive astrocytes by inhibiting the ROCK/NF-κB/C3 signaling pathway, thereby enhancing functional recovery following SCI. Fan et al. found that LPAR6 was predominantly expressed in spinal astrocytes, and the knockout of the LPAR6 gene reduced mechanical and thermal pain in mice undergoing CCI surgery [83]. Y-27632 increased the pain thresholds in the CCI mice by reducing the expression of C3 and p-NF-κB p65 and increasing the expression of S100A10 in the spinal cord. Mechanically, they found LPAR6 activated the NF-κB signal pathway and the A1 phenotype through ROCK2 in primary astrocytes. Consequently, the LPAR6/ROCK2/NF-κB/C3 signaling pathway, which mediates the transformation of astrocytes into a pro-inflammatory phenotype, is of great importance in pain.

### 6.1.3 Overproduction of cytokines

Deubiquitinating enzymes (DUBs) are important for counteracting the effects of ubiquitination, thereby governing protein function and stability. A study conducted by Li et al. demonstrated that ubiquitin-specific peptidase 53 (USP53) exacerbated neuropathic pain induced by CCI and augmented the levels of pro-inflammatory cytokines (e.g., IL-1β, IL-6, and TNF-α) in the spinal cord [51]. This outcome was achieved through the activation of the FK506 binding protein 51 (FKBP51)/RhoA/ROCK signaling pathway. Another study disclosed that preventive intrathecal administration of C3 exoenzyme or Y27632 effectively suppressed the release of spinal pro-nociceptive cytokines TNF-α and IL-1β in mice after intraplantar injection of LPS [20]. Furthermore, Li et al. identified that the expression of Erythropoietin-producing hepatocyte A1 Ephrin type-A receptor 1 (EphA1) was upregulated in the spinal cord in mice CCI model. Knockdown of EphA1 alleviated CCI-induced hyperalgesia, decreased the expression of pro-inflammatory cytokines (e.g., IL-1β, IL-6, and TNF-α), and increased the level of the anti-inflammatory cytokines (e.g., IL-10) [22]. It was also reported that EphA1 overexpression led to an increase in CXCL12 expression and significantly enhanced the enrichment of CXCR4, which interacts with CXCL12 [84]. The study further demonstrated that treatment with the CXCR4 antagonist AMD3100 and the ROCK2 inhibitor Fasudil could reverse exogenous EphA1-induced neuropathic pain in CCI mice, accompanied by a downregulation of pro-inflammatory cytokines (e.g., IL-1β, IL-6, and TNF-α) and an upregulation of anti-inflammatory cytokines (e.g., IL-10). The findings implied that the inhibition of the CXCR4/RhoA/ROCK2 pathway mitigated the effect of EphA1 on neuropathic pain and spinal inflammation. Additionally, intraplantar injection of LPS caused hyperalgesia and increased the production of TNF-α and IL-1β in the spinal cord, which were inhibited by preventive intrathecal treatment with either C3 exoenzyme or Y27632 [20]. Overall, this body of evidence indicates the crucial role of the Rho/ROCK signaling pathway in regulating cytokine production in the spinal cord.

### 6.2 RhoA/ROCK signaling and neuronal hyper-excitability

Wide dynamic range (WDR) neurons, a subtype of nociceptive neurons, are situated in the deep layers of the spinal dorsal horn and demonstrate increased sensitivity across diverse pain models. Monitoring the activity of WDR neurons offers an objective approach to investigate nociceptive modulation [85]. A study has indicated that the inhibition of ROCK using Fasudil (10 mg/kg, i.v.) significantly diminished both the spontaneous and evoked firing of WDR neurons in the spinal cords of SNL rats [41]. This indicates that RhoA/ROCK signaling contributes to central sensitization by augmenting the excitability of WDR neurons in the spinal dorsal horn. Furthermore, PKCγ plays a crucial role in the sensitization of dorsal horn neurons and is involved in increased pain sensitivity under multiple pain conditions [86, 87]. Inoue et al. noticed that the up-regulated expression of PKCγ, induced by both lysophosphatidic acid (LPA) and nerve injury, was prevented in mice lacking the LPA1 receptor, as well as in those treated with C3 exoenzyme [36]. These results suggested that receptor-mediated LPA signaling led to the up-regulation of spinal PKCγ through the activation of the RhoA/ROCK pathway, which is vital in the development of allodynia and hyperalgesia following SNI. An elevated expression of c-fos is associated with spinal neuronal sensitization. Wang et al. found that intrathecal administration of C3 exoenzyme or Y27632 alleviated LPS-induced hyperalgesia and inhibited the increase in spinal c-fos expression [20]. It has been reported that BDNF/CREB pathway regulates neuronal activity [88]. In MeHg-induced neuropathic pain rats, Fasudil inhibited the activation of dorsal horn neurons, as evidenced by the reduced levels of p-CREB and BDNF [25]. Additionally, the chemokine receptor CXCR4 is expressed in both spinal neurons and glial cells, which is implicated in the development and maintenance of neuropathic pain by promoting neuroinflammation and enhancing neuronal excitability [89, 90]. Xu et al. discovered that the intrathecal delivery of the CXCR4 inhibitor Plerixafor (AMD3100) or the ROCK2 inhibitor Fasudil abolished the TCI-induced increases in p-RhoA and p-ROCK2 expression in the spinal cord [21]. This research suggested that spinal RhoA/ROCK2 in neurons may be a key downstream target for CXCR4-mediated neuronal sensitization in bone cancer pain. Glutaminase (GLS) is a kay enzyme that converts glutamine into glutamate, a neurotransmitter that initiates the development of pain by enhancing the excitability of neurons during pain transmission [91]. The study indicated that fasudil elevated the mechanical pain threshold and decreased GLS1 expression in the spinal cords of rats suffering from CPSP [31]. Besides, it is widely acknowledged that increased synaptic plasticity significantly contributes to neuronal hyperexcitability [92]. P2Y12 knockout mice showed reduced nociceptive behaviors and a facilitation of miniature excitatory postsynaptic currents (mEPSCs) in spinal lamina II neurons after SNL [17]. Furthermore, a P2Y12 antagonist inhibited the activation of the RhoA/ROCK2/p38MAPK signaling pathway induced by SNL. As a result, the authors postulated that P2Y12 in microglia activated the RhoA/ROCK/p38MAPK signaling cascade, promoted the release of pro-inflammatory factors, and subsequently enhanced excitatory synaptic responses in the dorsal horn after nerve injury. Additionally, several investigations have shown that increased neurite outgrowth is related to an amplified nociceptive response [93]. The activation of the NO/cGMP/PKG signaling cascade in neurons leads to the phosphorylation and subsequent inactivation of the actin depolymerizing factor cofilin, ultimately resulting in enhanced actin stability and neurite outgrowth [71]. Y-27632 significantly lessened the phosphorylation of cofilin that was induced by the PKG-I activator 8-Br-cGMP in primary neurons. Moreover, NO-synthase inhibitor l-NAME and the ROCK inhibitor Y-27632 reduced cofilin phosphorylation in the spinal cord and alleviated zymosan-induced thermal hyperalgesia. The study indicated the NO/cGMP/PKG signaling regulated neurite outgrowth through its downstream RhoA/ROCK/cofilin pathway and thereby mediated nociception. However, the activation of the RhoA/ROCK pathway has been shown to cause the collapse of growth cones and prevent the extension of neurites [94, 95]. Slit is a key extracellular matrix protein crucial for guiding axonal growth and branching during nervous system development [96]. Evidence showed that sarcoma implantation led to an elevation of Slit2 levels in the spinal cord, accompanied by decreases in Robo1 and RhoA [30]. On the contrary, the knockdown of Slit2 increased the levels of Robo1 and RhoA, inhibited excitatory synaptogenesis, and alleviated bone cancer pain. This study suggested that the upregulation of Slit2 might contribute to mechanical allodynia after carcinoma implantation, likely by enhancing excitatory synapse formation through the suppression of Robo1 and subsequent removal of the RhoA-mediated inhibition of neurite outgrowth. RhoA inhibition facilitated neurite outgrowth, which could potentially contribute to pain possibly due to cytoskeletal ROCK substrates other than cofilin regulators. Myristoylated alanine-rich C-kinase substrate (MARCKS) plays a role in the trafficking of synaptic vesicles and the release of neurotransmitters in the CNS. Recent findings proposed that MARCKS phosphorylation by ROCK could trigger neurotransmitter release by reorganizing F-actin in the active zone of the presynaptse [97]. Intrathecal administration of Y27632, simvastatin, or H-1152 reduced pain in mice subjected to SNL, injection of formalin, and spinal nerve transection (SNT) by preventing the increase in phosphorylated MARCKS at Ser159 in the spinal cord [16, 45]. Notably, isoprenylation, a process catalyzed by farnesyl transferase and geranylgeranyl transferase, is crucial for the translocation of RhoA from the cytosol to the plasma membrane and the promotion of subsequent intracellular signaling [37]. Ohsawa et al. showed that blocking geranylgeranyl transferase I in the spinal cord with GGTI-2133 alleviated thermal hyperalgesia and mechanical allodynia in mice with partial SNL. At the same time, it also reduced the phosphorylation of the MARCKS protein in the ipsilateral spinal dorsal horn. These results suggested that neuropathic pain may occur due to the phosphorylation of the MARCKS protein, which is triggered by the activation of RhoA/ROCK signaling following geranylgeranylation [98]. Overall, these findings show that RhoA/ROCK signaling enhances spinal nociceptive responses by increasing neuronal excitability.

### 6.3 Other mechanisms of RhoA/ROCK signaling in spinal pain processing

NO serves as a crucial signaling molecule that influences nociception in both the peripheral and CNS [99, 100]. At the spinal cord level, evidence shows that a low concentration of NO-donors can bring about antinociceptive effects, whereas a high concentration tends to lower the nociceptive threshold [101]. Intrathecal injection of H-1152 alleviated neuropathic pain and remarkably suppressed the upregulation of NO production and the increase in phosphorylated MARCKS in the superficial layers of the spinal cord of nerve-transected mice [45].

**Figure 3.**
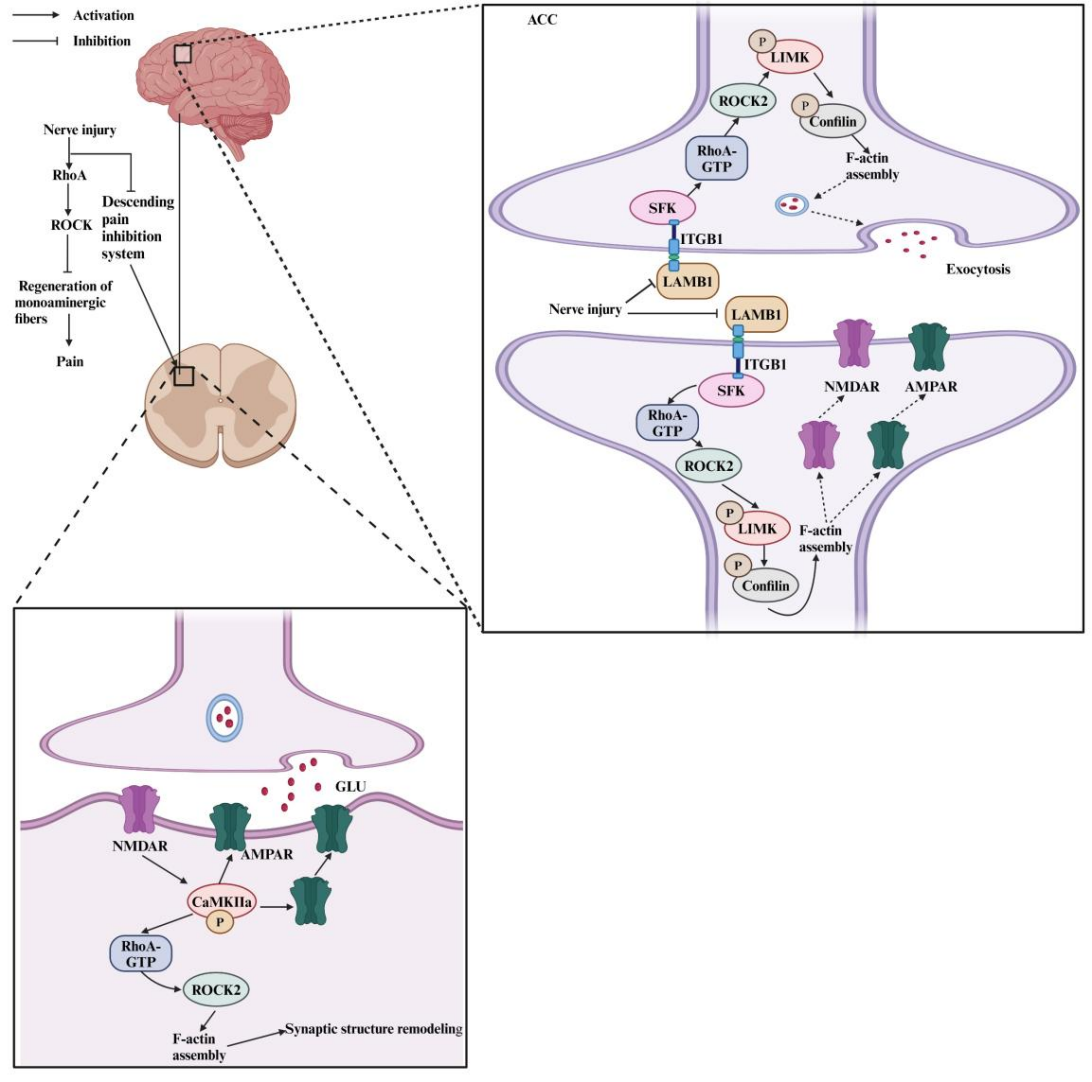
**The supraspinal mechanisms of RhoA/ROCK signaling in pain**. RhoA: Ras homolog gene family member A; ROCK: Rho-associated coiled-coil-containing protein kinase; CaMKIIa: Calcium/calmodulin-dependent protein kinase type IIa; NMDAR: N-methyl-D-aspartate receptor; AMPAR: α-amino-3-hydroxy-5-methyl-4-isoxazole-propionic acid receptor; LIMK: LIM kinase; LAMB1: laminin β1; ITGB1: integrin β1; SFK: Src-family non-receptor tyrosine kinase; GLU: glutamate. The figure is created in https://www.biorender.com.

This research suggested a close link between the NO generated due to the activation of the RhoA/ROCK pathway and the increase of phosphorylated MARCKS following nerve injury. Conversely, Ohsawa et al. indicated that simvastatin reduced thermal hyperalgesia and mechanical pain in diabetic mice by enhancing the levels of NO metabolites in the spinal cord [29]. This effect was achieved through upregulation of endothelial NO synthase (eNOS) protein expression, which was mediated by the inhibition of the RhoA/ROCK signaling pathway. The RhoA/ROCK pathway seems to be involved in modulating spinal sensitization by affecting NO production. However, more research is needed to gain a better understanding of how RhoA/ROCK pathway regulates NO production and to clarify the role of NO in pain.

## 7. Supraspinal mechanisms of RhoA/ROCK signaling in pain

The anterior cingulate cortex (ACC) plays a crucial role in processing and perceiving pain within the brain. It is well established that both structural and functional synaptic plasticity in the ACC provides the cellular basis for the onset and progression of neuropathic pain [92]. Notably, a significant down-regulation of the extracellular matrix protein laminin β1 (LAMB1) was detected in the ACC of mice subjected to SNI [102]. Moreover, silencing LAMB1 in the ACC resulted in increased pain sensitivity. Detailed mechanistic studies revealed that the loss of LAMB1 triggered F-actin polymerization and disrupted the actin cytoskeleton by activating the RhoA/LIMK/cofilin pathway through Src-dependent mechanisms, resulting in abnormal spine remodeling in ACC pyramidal neurons. Previous research has indicated that the trafficking of the α-amino-3-hydroxy-5-methyl-4-isoxazole-propionic acid receptor (AMPAR) membrane is dependent upon the integrity of the actin cytoskeleton. Additionally, acting polymerization has been linked to the transport of presynaptic vesicles and the release of neurotransmitters [103-105]. Li et al. further indicated that down-regulation of LAMB1 induced actin cytoskeleton rearrangement via the RhoA/LIMK/cofilin pathway following peripheral nerve injury, resulting in an increase in the probability of presynaptic neurotransmitter release and synaptic trafficking of the AMPAR subunit GluR1 and the NMDAR subunit NR2A within pyramidal neurons. Ultimately, the potentiated synaptic function contributed to pain hypersensitivity [102] (Fig 3).

Nociceptive transmission from the spinal dorsal horn is modulated by descending monoaminergic systems, which encompass serotonergic, noradrenergic, and dopaminergic pathways. Pain has been associated with dysfunction of these descending pain inhibitory pathways [106-108]. Activation of the RhoA/ROCK signaling pathway has been found to hinder neurite growth and inhibit sprouting. Consequently, targeting this pathway may enhance axon regeneration and support functional recovery following SCI [109, 110]. Evidence indicated that the knockdown of RhoA with siRhoA alleviated allodynia at 6 to 8 weeks post SCI, likely due to the promotion of serotonergic axonal regrowth caudal to the injury site [28]. Furthermore, intrathecal administration of Y-27632 has been found to stimulate the sprouting of intact supraspinal monoaminergic fibers, which may be the mechanism by which cold hyperalgesia is alleviated after dorsal rhizotomy [33]. Additionally, LPM580098, an innovative monoamine reuptake inhibitor, has been shown to diminish mechanical allodynia, thermal hyperalgesia, and hyperexcitability of WDR neurons in SNL rats [27]. The proposed analgesic mechanism of LPM580098 involved augmenting presynaptic neurotransmitter release, such as serotonin, norepinephrine, and dopamine, which activated descending inhibitory systems and decreased the release of excitatory neurotransmitters in the spinal dorsal horn. Subsequently, this process inhibited the postsynaptic amplification of nociception via the NR2B/CaMKIIα/GluR1 signaling pathway. Studies have shown that the size and density of dendritic spines in the superficial layers of the spinal cord increase in various neuropathic pain models, highlighting their significance in nociception [111]. Accumulating evidence indicates that RhoA/ROCK signaling affects the morphology and functionality of dendritic spines by modulating the organization of the actin cytoskeleton [112, 113]. Consequently, Li et al. further indicated that LPM580098 exerts its analgesic effects by mitigating postsynaptic dendritic spine remodeling through down-regulating the RhoA/ROCK signaling pathways [27]. Overall, RhoA/ROCK signaling appears to play a significant role in pain regulation by interacting with the descending pain inhibition system.

## 8. Summary and Future Perspective

Our work stands as the first to deliver a comprehensive overview of the role of RhoA/ROCK signaling in pain, as well as the mechanisms through which it modulates pain across different levels of the neuroaxis. Additionally, we explore the therapeutic potential of pharmacological inhibitors that target the RhoA/ROCK pathway in the treatment of pain (Table 1).

The study of pain to identify drug targets and mechanisms of action has relied extensively on animal models for centuries. Rat models are used in pain research due to their physiological and pain pathway similarities to humans [114]. Various animal pain models have been developed to replicate different pain conditions in humans [115, 116]. Numerous novel mechanism-based therapies have proven effective in both animal models and relevant patient populations. However, inherent differences among species indeed exist. The complexity of the human central nervous system, characterized by its developed cerebral cortex and more intricate neural connections, may lead to different pain processing. Besides, pain is often inferred from behavioral changes in animals, whereas humans can verbally report their pain intensity and duration, which is a crucial aspect of pain assessment and treatment evaluation. Moreover, human pain is a complex, heterogeneous disease state and influenced by a variety of modulatory factors, such as sex, psychological, social, and environmental factors [117]. Notably, patients often have comorbidities that can influence pain perception and treatment outcomes. Previous studies have highlighted four key factors that influence research outcomes: the selection of subject (model organism), the choice of assay (pain-inducing injury), the laboratory environment, and the selection of outcome measures [118], which may be hampering translational success. In summary, refining several aspects of animal models of pain can facilitate better clinical translation.

**Table 1:** Summary of the therapeutic potential of pharmacological inhibitors of RhoA/ROCK signaling in pain.

Class of compound	Compound	Model	Treatment strategy	Effects	Mechanisms	Expression	Ref.
Inhibition of the isoprenylation of RhoA	simvastatin	Formalin-induced inflammatory pain mice	Simvastatin (20, 40 mg/kg, i.p.) was administered 1h before formalin injection	licking and biting time (phase II) ↓	RhoA/ROCK signaling pathway activation↓	Spinal cord	[39]
		**STZ-induced diabetic neuropathic pain mice**	Simvastatin (2.5, 5, 10 and 20 mg/kg, i.p.) was administered daily for 7 consecutive days starting from one week after STZ injection	TFL, PWT↑	Membrane-bound RhoA↓, eNOS, NOx↑	Spinal cord	[30]
		**PSNL-induced neuropathic pain mice**	Simvastatin (5 μg, i.t. or 5, 10, 20 mg/kg, i.p.) was administered for 10 consecutive days starting 3 days before nerve ligation	PWL, PWT↑	RhoA/ROCK signaling pathway activation↓, p-MARCKS↓, astroglial and microglial activation↓	Spinal cord	[18]
		**CCI-induced neuropathic pain rat**	Simvastatin (10 μg, i.t.) was administered once daily for 7 days after CCI	PWL, PWT↑	Inhibition of the RhoA/LIMK/cofilin pathway	DRG	[41]
		**Formalin-induced inflammatory pain rat**	Simvastatin (0.1, 1, 10 μg, i.t.) was administered 30 min before formalin injection and once daily for 7 days after injection	NOF (phase II) ↓, PWT↑	RhoA activation, microglia activation↓, p-p38 MAPK↓	Spinal cord	[27]
		**Formalin-induced inflammatory pain mice**	Simvastatin (0.5, 5, 50 nmol, i.t.) was administered 30 min before formalin injection	licking and biting time (phase II) ↓	RhoA/ROCK signaling pathway activation↓	Spinal cord	[40]
**Inhibition of the geranylgeranylation of RhoA**	GGTI-2133	PSNL-induced neuropathic pain mice	GGTI-2133 (0.001, 0.01, 0.1 nmol, i.t.) was administered 1 h before the nerve injury and once daily for 7 days after injure	PWL, PWT↑	p-MARCKS↓	Spinal cord	[97]
**RHOA inhibitor**	C3 exoenzyme	STZ-induced diabetic neuropathic pain mice	C3 exoenzyme (1, 3 and 10 pg, i.t.) was administered for one week after the STZ injection	TFL, PWT↑	Inhibition of RhoA/ROCK signaling pathway	Spinal cord	[30]
		Formalin-induced inflammatory pain mice	C3 exoenzyme (10 pg, i.t.) was administered 30 min before formalin injection	licking and biting time (phase II) ↓	p-p38 MAPK, IL-1β↓	Spinal cord	[21]
		**TCI-induced bone cancer pain rat**	C3 exoenzyme (10 pg, i.t.) was administered on day 9 after TCI	PWT↑	/	Spinal cord	[20]
		**LPS-induced inflammatory pain mice**	C3 exoenzyme (10 pg, i.t.) was administered 30 min before LPS injection	PWL, PWT↑	TNF-α, IL-1β, c-fos↓	Spinal cord	[22]
		**TCI-induced bone cancer pain rat**	C3 exoenzyme (300 pg, i.t.) was administered on day 16 after TCI	PWT↑	P2X3R-mediated calcium influx in DRG neurons↓	DRG	[35]
		**PSNL-induced neuropathic pain mice**	C3 exoenzyme (10 pg, i.t.) was administered 1h before or 1h after the nerve injury	PWT↑	Demyelination, PKCγ, Cavα2δ-1↓, MBP, PMP22↑	Spinal cord and DRG	[37]
	**siRhoA**	SCI-induced neuropathic pain rat	siRhoA (1 μg) was administered 30 min before injury at the injury epicenter, 2 mm rostral and caudal to the epicenter	PWT↑	Activated macrophages↓, serotonergic axonal regrowth↑	Spinal cord	[29]
ROCK inhibitor	Y-27632	STZ-induced diabetic neuropathic pain mice	Y-27632 (0.1, 1 and 10 nmol, i.t.) was administered for one week after the STZ injection	TFL, PWT↑	Inhibition of RhoA/ROCK signaling pathway	Spinal cord	[30]
		**Formalin-induced inflammatory pain mice**	Y-27632 (10 nmol, i.t.) was administered 30 min before formalin injection	licking and biting time (phase II) ↓	p-p38 MAPK,IL-1β, number of activated microglial↓	Spinal cord	[21]
		**LPS-induced inflammatory pain mice**	Y27632 (10 nmol, i.t.) was administered 30 min before LPS injection	PWL, PWT↑	TNF-α, IL-1β, c-fos↓	Spinal cord	[22]
		**TCI-induced bone cancer pain rats**	Y27632 (48 μg, i.t.) was administered on day 16 after TCI	PWT↑	P2X3R-mediated calcium influx in DRG neurons↓	DRG	[35]
		**PSNL-induced neuropathic pain mice**	Y27632 (3 μg, i.t.) was administered three times a day from 1 day before to 5 days after SNL	PWL, PWT↑	p-p38MAPK↓	Spinal cord	[19]
		**PSNL-induced neuropathic pain mice**	Y27632 (10 nmol, i.t.) was administered 30 min before nerve ligation and up to 7 days after nerve ligation	PWT↑	p-MARCKS, astroglial activation↓	Spinal cord	[18]
		**CCI-induced neuropathic pain rat**	Y-27632 (48 μg, i.t.) was administered once daily for 7 days after CCI	PWL, PWT↑	Inhibition of the RhoA/LIMK/cofilin pathway	DRG	[41]
		**Zymosan-induced inflammatory pain rat**	Y-27632 (100, 625 μg/kg, i.t.) was administered for 5 days before zymosan injection.	PWL↑	p-cofilin↓	Spinal cord	[72]
		**SNI-induced neuropathic pain mice**	Y-27632 (10 nmol, i.t.) was administered 1 h before nerve injury	PWT↑	Demyelination, PKCγ, Cavα2δ-1↓, MBP, PMP22↑	/	[37]
		**Carrageenan-induced inflammatory pain rat**	Y27632 (*500 μg*) was administered 5 min before carrageenan to *rat hind paw*	Nociceptive threshold↑	/	/	[45]
		**C7/8 rhizotomy-induced cold hyperalgesia rat**	Y-27632 (48 μg, i.t.) was administered for 10 days after surgery	Time of withdrawal, biting or licking ↓ (after acetone squirted onto the palmar surface)	Sprouting of monoaminergic axons↑	Spinal cord	[34]
		CCI-induced neuropathic pain mice	Y-27632 (0.1 mg/kg/d, i.p.) was administered	PWT, TWL↑	C3, p-NF-κB p65/NF-κB p65↓, S100A10↑	Spinal cord	[83]
	Fasudil (HA-1077)	TCI-induced bone cancer pain rat	Fasudil (20 μg, i.t.) was administered from 5 to 7 days or from 12 to 14 days after TCI	PWL, PWT↑	/	Spinal cord	[23]
		**CPSP rat**	Fasudil (10 mg/kg, i.p.) was administered for 7 days after surgery	PWT↑, CAS pain ↓	GLS1↓	Spinal cord	[32]
		**CCI-induced neuropathic pain rat**	Fasudil (3, 10, 30 mg/kg, i.p.) was administered on day 14 after surgery	PWT↑	/	/	[42]
		**SNL-induced neuropathic pain rat**	Fasudil (3, 10, 30 mg/kg, i.p.) was administered on day 7 after surgery	PWT↑	/	/	[42]
		**Capsaicin-induced inflammatory pain rat**	Fasudil (3, 10, 30 mg/kg, i.p.) was administered after capsaicin injection	NOF↓, PWT↑	/	/	[42]
		**MIA-induced osteoarthritis pain rat**	Fasudil (3, 10, 30 mg/kg, i.p.) was administered on day 20 after monoiodoacetate injection	CFmax↑	/	/	[42]
		**Carrageenan-induced inflammatory pain rat**	Fasudil (3, 10, 30 mg/kg, i.p.) was administered 1.5 h after carrageenan injection	PWL↑	/	/	[42]
		**MIA-induced osteoarthritis pain rat**	Fasudil (3, 30 mg/kg, o.p.) was administered from day 21 to 28 after monoiodoacetate injection	Weight distribution↓	/	/	[43]
		**AIA-induced osteoarthritis pain rat**	Fasudil (30 mg/kg, o.p.) was administered from day 18 to 21 after adjuvant	PWT↑	/	/	[43]
		MeHg-induced neuropathic pain rat	Fasudil (3 mg/kg, s.c.) was performed from day 21 to 63 after MeHg administration	Avoidance threshold/body weight↑	TNF-α, iNOS, IL-1β, IL-6, p-NF-κB p65/NF-κB p65, p-CREB, BDNF↓, arginase-1 and IL-10↑	Spinal cord	[25]
	**Ripasudil**	SCI-induced neuropathic pain rat	Ripasudil (24, 240 nmol, i.t.) was administered from just before SCI to 3 days after SCI	PWT↑	p-p38 MAPK, the immunoreactivity of Iba1↓	Spinal cord	[44]
	H1152	SNI-induced neuropathic pain rat	H1152 (12, 24, 240 pmol, i.t.) was administered from 1 to 3 days after nerve injury	PWT↑	p-p38 MAPK↓, inhibition of morphology of activated microglia	Spinal cord	[47]
		**Formalin-induced inflammatory pain mice**	H1152 (10, 100 ng, i.t.) was administered 10 min before formalin injection	Licking and biting time (second II) ↓	p-MARCKS, NO synthase activity↓	Spinal cord	[46]
		**SNT-induced neuropathic pain mice**	H1152 (10, 100 ng, i.t.) was administered on day 7 after nerve transection	PWT↑	p-MARCKS, NO synthase activity↓	Spinal cord	[46]
	AS1892802	AIA-induced osteoarthritis pain rat	AS1892802 (1 mg/kg, o.p.) was administered from day 18 to 21 after adjuvant	PWT↑	/	/	[43]
		**MIA-induced osteoarthritis pain rat**	AS1892802 (0.1, 0.3, 1 mg/kg, o.p.) was administered from day 21 to 28 after monoiodoacetate injection	Weight distribution↓	/	/	[43]
		**MIA-induced osteoarthritis pain rat**	AS1892802 (3 μg, i.a.) was administered from day 21 to 28 after monoiodoacetate injection	Weight distribution↓	/	/	[43]
		**MIA-induced osteoarthritis pain rat**	AS1892802 (0.03, 0.3, 3 μg, i.a.) was administered twice a week for three weeks/AS1892802 (1, 3.2, 10 mg/kg, p.o.) administered once a day for 3 weeks starting the day of the monoiodoacetate injection	Weight distribution↓	/	Distal ends of femur condyles	[48]
		**MIA-induced osteoarthritis pain rat**	AS1892802 (0.1, 0.3 mg/kg, p.o.) was administered on day 21 or twice a day between 14 and 28 days after monoiodoacetate injection	weight distribution↓	/	/	[49]
		**STZ-induced diabetic neuropathic pain rat**	AS1892802 (0.03, 0.1, 0.3, 1 mg/kg, p.o.) was administered once daily between 21 and 49 days after STZ injection	PWT↑	/	/	[49]
	**miR-488-3p**	CCI-induced neuropathic pain rat	LV-miR-488-3p (3.0×10^8^ TU/Ml, 5 μl, i.t.) was administered every 24 h before 72 h of injure	PWL, PWT↑	TNF-α, IL-1β, IL-6↓, IL-4, IL-10↑	DRG	[53]

Abbreviations: CCI: chronic constriction injury; SNI: spared nerve injury; PSNL: partial spinal nerve ligation; SCI: spinal cord injury; SNT: spinal nerve transection; CFA: Complete Freund’s adjuvant; AIA: adjuvant-induced arthritis; MIA: monoiodoacetate-induced arthritis; MeHg: methylmercury; CPSP: chronic post-thoracotomy pain; GLS: glutaminase; CAS: cold acetone stimulus; DRG: dorsal root ganglion; eNOS: endothelial nitric oxide synthetase; NOx: nitric oxide metabolites; IL-1β: interleukin 1beta; IL-6: interleukin 6; TNF-α: tumor necrosis factor α; i.p.: intraperitoneally; i.t.: intrathecally; p.o.: peros; s.c.:subcutaneously; LPS: lipopolysaccharide; PWL: paw withdrawal latency; PWT: paw withdrawal threshold; TWL: Thermal withdrawal latency; TFL: tail-flick latency; NOF: number of flinches; CFmax: maximum compressive force; i.a.: intra-articularly; STZ: streptozotocin; TCI: tumor cell implantation; NF-κB: nuclear factor kappa B; CREB: CRE binding protein; BDNF: brain-derived neurotrophic factor; iNOS: inducible nitric oxide synthase; P2X3R: purinergic P2X3 receptor; PKCγ: γ-isoform of protein kinase C; MARCKS: myristoylated alanine-rich protein kinase C substrate; RhoA: Ras homolog gene family member A; ROCK: Rho-associated coiled-coil-containing protein kinase; LIMK: LIM kinase; MAPK: mitogen-activated protein kinases; ↑: upregulated; ↓: downregulated.

There are several limitations that warrant attention. First, none of the RhoA/ROCK inhibitors mentioned in this review are specific inhibitors, which may induce off-target effects. Current pharmacological inhibitors targeting the RhoA/ROCK signaling pathway exhibit low potency in comparison to clinically approved kinase inhibitors and inhibit additional kinases as well [119], which somewhat constrains the interpretation of results. The creation of more selective and potent RhoA/ROCK inhibitors, specifically designed for use as therapeutic agents in humans, remains a significant challenge.

Second, despite their strong homology, ROCK isoforms demonstrate distinct and nonredundant roles. Existing research has yet to unveil the varying functions of ROCK isoforms in pain regulation. KD-025 is the first selective inhibitor targeting ROCK2 specifically [120]. Progress in developing isoform-selective ROCK inhibitors, along with the generation of cell- and tissue-specific ROCK1 and ROCK2 knockout mice, is expected to be crucial for understanding the isoform-specific roles of ROCK in pain mechanisms.

Third, The RhoA/ROCK signaling pathway forms a highly intricate network. The RhoA/ROCK pathway plays a complex and multifaceted role in the regulation of nociceptive sensitization. There was abundant evidence suggesting that the predominant effect associated with RhoA/ROCK signaling activation is pro-nociception. However, several studies indicated that RhoA/ROCK signaling is implicated in antinociceptive mechanisms [30, 72-74]. Additional research is essential to further elucidate the functions and specific mechanisms of RhoA/ROCK signaling in pain conditions.

Fourth, despite the promising therapeutic effects of RhoA/ROCK inhibitors in animal pain models, there are currently no clinical trials available to assess their efficacy in humans. Additional research and clinical studies are required to assess the therapeutic potential and optimal use of RhoA/ROCK inhibitors for pain management. Notably, several drugs targeting the RhoA/ROCK signaling pathway have already been implemented in clinical settings. The ROCK inhibitor Fasudil is approved for clinical use aimed at treating and preventing cerebral ischemia in China and Japan [121]. Simvastatin is an FDA-approved lipid-lowering agent [122]. These preclinical studies hold promises for rapid translation into clinical trials, exploring the analgesic effects of these inhibitors in pain management. While blocking the RhoA/ROCK pathway holds significant therapeutic potential for pain and other conditions, the potential side effects must be carefully weighed. The RhoA/ROCK pathway is involved in numerous physiological processes. The high rate of embryonic and perinatal mortality observed in mice with complete homozygous deletion of ROCK isoforms highlights the critical role of this pathway in essential cellular functions [119]. Previous research demonstrated that the RhoA/ROCK pathway was essential for the survival of both developing and mature neurons and its inhibition could potentially result in motor deficits [123, 124]. Furthermore, systemic inhibition of this pathway can lead to side effects, including low blood pressure and compromised wound healing [125, 126], as it plays a crucial role in modulating vascular smooth muscle tension and cell migration and proliferation. Therefore, potential adverse effects warrant careful consideration, as they may restrict the clinical application of RhoA/ROCK inhibitors. Moreover, given the potentially narrow therapeutic window for RhoA/ROCK inhibitors, studies on their pharmacokinetics, optimal dosing, and long-term safety in pain treatment are essential. Notably, the lack of an orally administered formulation limits the long-term clinical application of Fasudil. Fortunately, a Phase I Trial conducted by Wolff et. al indicated that oral fasudil appeared generally safe and well tolerated in the studied cohort [127], which provides a foundation for future trials exploring the use of fasudil in chronic pain, where long-term oral administration is necessary.

Fifth, effective delivery of RhoA/ROCK inhibitors to the target site remains a significant challenge. The development of targeted delivery systems is necessary to enhance therapeutic efficacy while minimizing systemic exposure. The localized delivery of the C3 protein faces challenges, including poor cell permeability and a limited duration of action. Emerging evidence shows that gene therapy is a promising method for persistent treatment of many diseases. It was reported that viral vector-mediated delivery of C3-expressing constructs provided long-lasting effects and potentially bypassed the invasiveness of direct injection [128, 129]. This method has the potential to address the limitations associated with protein-based delivery and improve therapeutic outcomes.

Finally, a more comparative analysis that provides context for the efficacy and safety of RhoA/ROCK inhibitors relative to current options is necessary. A prior investigation assessed the analgesic properties of tramadol, diclofenac, and the ROCK inhibitors AS1892802 and fasudil in two rat models of chronic arthritis [42]. The results showed that oral administration of tramadol (30 mg/kg), AS1892802 (1 mg/kg), and fasudil (30 mg/kg) produced analgesic effects in both MIA and AIA rats. In contrast, diclofenac exhibited an analgesic effect only in AIA rats, with the minimum effective dose being 1 mg/kg, and the effect was dose-dependent. The potency of tramadol and fasudil was weaker than that of AS1892802. Although ROCK inhibitors are commonly used as antihypertensive agents, AS1892802 did not show significant hypotensive effects, even at doses as high as 3 mg/kg. Additionally, nonsteroidal anti-inflammatory drugs (NSAIDs) can lead to gastrointestinal toxicity, and tramadol may result in side effects such as nausea, vomiting, dizziness, and abnormal locomotor activity. Conversely, AS1892802 exhibited a favorable safety profile. The study indicated that AS1892802 possessed a potent analgesic effect in rats with pain under therapeutic dosing, without inducing the severe side effects commonly associated with traditional analgesics.

While targeting the RhoA/ROCK signaling pathway holds great promise as a therapeutic strategy for pain management, several limitations need to be overcome. Future research should prioritize enhancing the specificity, safety, and delivery methods of these inhibitors, alongside conducting comprehensive clinical trials to confirm their effectiveness in patients.

## References

[1] TreedeRD, RiefW, BarkeA, AzizQ, BennettMI, BenolielR, et al. (2019). Chronic pain as a symptom or a disease: the IASP Classification of Chronic Pain for the International Classification of Diseases (ICD-11). Pain, 160:19-27.30586067 10.1097/j.pain.0000000000001384

[2] CohenSP, VaseL, HootenWM (2021). Chronic pain: an update on burden, best practices, and new advances. Lancet, 397:2082-2097.34062143 10.1016/S0140-6736(21)00393-7

[3] LiuD, ZhouY, PengY, SuP, LiZ, XuQ, et al. (2018). Endoplasmic Reticulum Stress in Spinal Cord Contributes to the Development of Morphine Tolerance. Front Mol Neurosci, 11:72.29559889 10.3389/fnmol.2018.00072PMC5845556

[4] KaibuchiK, KurodaS, AmanoM (1999). Regulation of the cytoskeleton and cell adhesion by the Rho family GTPases in mammalian cells. Annu Rev Biochem, 68:459-486.10872457 10.1146/annurev.biochem.68.1.459

[5] HagaRB, RidleyAJ (2016). Rho GTPases: Regulation and roles in cancer cell biology. Small GTPases, 7:207-221.27628050 10.1080/21541248.2016.1232583PMC5129894

[6] Kalpachidou T, Spiecker L, Kress M, Quarta S (2019). Rho GTPases in the Physiology and Pathophysiology of Peripheral Sensory Neurons. Cells, 8. 10.3390/cells8060591 PMC6627758 31208035

[7] WarnerH, WilsonBJ, CaswellPT (2019). Control of adhesion and protrusion in cell migration by Rho GTPases. Curr Opin Cell Biol, 56:64-70.30292078 10.1016/j.ceb.2018.09.003PMC6368645

[8] Martin-CamaraO, CoresA, Lopez-AlvaradoP, MenendezJC (2021). Emerging targets in drug discovery against neurodegenerative diseases: Control of synapsis disfunction by the RhoA/ROCK pathway. Eur J Med Chem, 225:113742.34388381 10.1016/j.ejmech.2021.113742

[9] IyerM, SubramaniamMD, VenkatesanD, ChoSG, RydingM, MeyerM, et al. (2021). Role of RhoA-ROCK signaling in Parkinson's disease. Eur J Pharmacol, 894:173815.33345850 10.1016/j.ejphar.2020.173815

[10] LuW, ChenZ, WenJ (2023). The role of RhoA/ROCK pathway in the ischemic stroke-induced neuroinflammation. Biomed Pharmacother, 165:115141.37437375 10.1016/j.biopha.2023.115141

[11] Etienne-MannevilleS, HallA (2002). Rho GTPases in cell biology. Nature, 420:629-635.12478284 10.1038/nature01148

[12] CherfilsJ, ZeghoufM (2013). Regulation of small GTPases by GEFs, GAPs, and GDIs. Physiol Rev, 93:269-309.23303910 10.1152/physrev.00003.2012

[13] Schmidt SI, Blaabjerg M, Freude K, Meyer M (2022). RhoA Signaling in Neurodegenerative Diseases. Cells, 11. 10.3390/cells11091520 PMC9103838 35563826

[14] LiuM, BiF, ZhouX, ZhengY (2012). Rho GTPase regulation by miRNAs and covalent modifications. Trends Cell Biol, 22:365-373.22572609 10.1016/j.tcb.2012.04.004PMC3383930

[15] NakagawaO, FujisawaK, IshizakiT, SaitoY, NakaoK, NarumiyaS (1996). ROCK-I and ROCK-II, two isoforms of Rho-associated coiled-coil forming protein serine/threonine kinase in mice. FEBS Lett, 392:189-193.8772201 10.1016/0014-5793(96)00811-3

[16] OhsawaM, IshikuraK, MutohJ, HisaH (2016). Involvement of inhibition of RhoA/Rho kinase signaling in simvastatin-induced amelioration of neuropathic pain. Neuroscience, 333:204-213.27457035 10.1016/j.neuroscience.2016.07.029

[17] YuT, ZhangX, ShiH, TianJ, SunL, HuX, et al. (2019). P2Y12 regulates microglia activation and excitatory synaptic transmission in spinal lamina II neurons during neuropathic pain in rodents. Cell Death Dis, 10:165.30778044 10.1038/s41419-019-1425-4PMC6379416

[18] HangLH, ShaoDH, ChenZ, SunWJ (2013). Spinal RhoA/Rho kinase signalling pathway may participate in the development of bone cancer pain. Basic Clin Pharmacol Toxicol, 113:87-91.23521814 10.1111/bcpt.12069

[19] WongSSC, LeeUM, WangXM, ChungSK, CheungCW (2019). Role of DLC2 and RhoA/ROCK pathway in formalin induced inflammatory pain in mice. Neurosci Lett, 709:134379.31323253 10.1016/j.neulet.2019.134379

[20] WangC, SongS, ZhangY, GeY, FangX, HuangT, et al. (2015). Inhibition of the Rho/Rho kinase pathway prevents lipopolysaccharide-induced hyperalgesia and the release of TNF-alpha and IL-1beta in the mouse spinal cord. Sci Rep, 5:14553.26416580 10.1038/srep14553PMC4586490

[21] XuH, PengC, ChenXT, YaoYY, ChenLP, YinQ, et al. (2020). Chemokine receptor CXCR4 activates the RhoA/ROCK2 pathway in spinal neurons that induces bone cancer pain. Mol Pain, 16:1744806920919568.32349612 10.1177/1744806920919568PMC7227150

[22] LiQ, LiR, ZhuX, ChuX, AnX, ChenM, et al. (2023). EphA1 aggravates neuropathic pain by activating CXCR4/RhoA/ROCK2 pathway in mice. Hum Cell, 36:1416-1428.37162645 10.1007/s13577-023-00911-9

[23] ZhangD, JingB, ChenZ, LiX, ShiH, ZhengY, et al. (2022). Ferulic acid alleviates sciatica by inhibiting peripheral sensitization through the RhoA/p38MAPK signalling pathway. Phytomedicine, 106:154420.36115115 10.1016/j.phymed.2022.154420

[24] LiX, ShiH, ZhangD, JingB, ChenZ, ZhengY, et al. (2023). Paeonol alleviates neuropathic pain by modulating microglial M1 and M2 polarization via the RhoA/p38MAPK signaling pathway. CNS Neurosci Ther, 29:2666-2679.37032648 10.1111/cns.14211PMC10401133

[25] FujimuraM (2022). Fasudil, a ROCK inhibitor, prevents neuropathic pain in Minamata disease model rats. Toxicol Lett, 371:38-45.36244566 10.1016/j.toxlet.2022.10.001

[26] ChenXY, LiK, LightAR, FuKY (2013). Simvastatin attenuates formalin-induced nociceptive behaviors by inhibiting microglial RhoA and p38 MAPK activation. J Pain, 14:1310-1319.23900131 10.1016/j.jpain.2013.05.011

[27] LiN, LiC, HanR, WangY, YangM, WangH, et al. (2019). LPM580098, a Novel Triple Reuptake Inhibitor of Serotonin, Noradrenaline, and Dopamine, Attenuates Neuropathic Pain. Front Pharmacol, 10:53.30837867 10.3389/fphar.2019.00053PMC6382704

[28] OtsukaS, AdamsonC, SankarV, GibbsKM, Kane-GoldsmithN, AyerJ, et al. (2011). Delayed intrathecal delivery of RhoA siRNA to the contused spinal cord inhibits allodynia, preserves white matter, and increases serotonergic fiber growth. J Neurotrauma, 28:1063-1076.21443453 10.1089/neu.2010.1568

[29] OhsawaM, AasatoM, HayashiSS, KameiJ (2011). RhoA/Rho kinase pathway contributes to the pathogenesis of thermal hyperalgesia in diabetic mice. Pain, 152:114-122.20980102 10.1016/j.pain.2010.10.005

[30] KeC, GaoF, TianX, LiC, ShiD, HeW, et al. (2017). Slit2/Robo1 Mediation of Synaptic Plasticity Contributes to Bone Cancer Pain. Mol Neurobiol, 54:295-307.26738857 10.1007/s12035-015-9564-9

[31] LiuZY, WangHT, TangJ, QinZS (2017). [RhoA/Rho-kinase contributes to chronic pain following thoracotomy by up-regulating glutaminase 1 expression in rat spinal dorsal cord]. Nan Fang Yi Ke Da Xue Xue Bao, 37:1358-1363.29070466 10.3969/j.issn.1673-4254.2017.10.12PMC6743961

[32] BuyukafsarK, YalcinI, KurtAH, TiftikRN, Sahan-FiratS, AksuF (2006). Rho-kinase inhibitor, Y-27632, has an antinociceptive effect in mice. Eur J Pharmacol, 541:49-52.16750189 10.1016/j.ejphar.2006.04.042

[33] RamerLM, BorisoffJF, RamerMS (2004). Rho-kinase inhibition enhances axonal plasticity and attenuates cold hyperalgesia after dorsal rhizotomy. J Neurosci, 24:10796-10805.15574730 10.1523/JNEUROSCI.3337-04.2004PMC6730209

[34] Wu JX, Yuan XM, Wang Q, Wei W, Xu MY (2016). Rho/ROCK acts downstream of lysophosphatidic acid receptor 1 in modulating P2X3 receptor-mediated bone cancer pain in rats. Mol Pain, 12. 10.1177/1744806916644929 PMC4956381 27094551

[35] ChanFK, ChungSS, NgIO, ChungSK (2012). The RhoA GTPase-activating protein DLC2 modulates RhoA activity and hyperalgesia to noxious thermal and inflammatory stimuli. Neurosignals, 20:112-126.22204965 10.1159/000331240

[36] InoueM, RashidMH, FujitaR, ContosJJ, ChunJ, UedaH (2004). Initiation of neuropathic pain requires lysophosphatidic acid receptor signaling. Nat Med, 10:712-718.15195086 10.1038/nm1060

[37] HoshinoY, GaucherEA (2018). On the Origin of Isoprenoid Biosynthesis. Mol Biol Evol, 35:2185-2197.29905874 10.1093/molbev/msy120PMC6107057

[38] OhsawaM, MutohJ, HisaH (2008). Mevalonate sensitizes the nociceptive transmission in the mouse spinal cord. Pain, 134:285-292.17764839 10.1016/j.pain.2007.04.031

[39] OhsawaM, MutohJ, YamamotoS, OnoH, HisaH (2012). Effect of spinally administered simvastatin on the formalin-induced nociceptive response in mice. J Pharmacol Sci, 119:102-106.22510521 10.1254/jphs.12007sc

[40] QiuY, ChenWY, WangZY, LiuF, WeiM, MaC, et al. (2016). Simvastatin Attenuates Neuropathic Pain by Inhibiting the RhoA/LIMK/Cofilin Pathway. Neurochem Res, 41:2457-2469.27216618 10.1007/s11064-016-1958-1

[41] Boyce-RustayJM, SimlerGH, McGaraughtyS, ChuKL, WensinkEJ, VasudevanA, et al. (2010). Characterization of Fasudil in preclinical models of pain. J Pain, 11:941-949.20338818 10.1016/j.jpain.2009.12.014

[42] YoshimiE, KumakuraF, HatoriC, HamachiE, IwashitaA, IshiiN, et al. (2010). Antinociceptive effects of AS1892802, a novel Rho kinase inhibitor, in rat models of inflammatory and noninflammatory arthritis. J Pharmacol Exp Ther, 334:955-963.20534789 10.1124/jpet.110.167924

[43] KishimaK, TachibanaT, YamanakaH, KobayashiK, OkuboM, MaruoK, et al. (2021). Role of Rho-associated coiled-coil containing protein kinase in the spinal cord injury induced neuropathic pain. Spine J, 21:343-351.32853793 10.1016/j.spinee.2020.08.011

[44] Paiva-LimaP, BakhleYS, FrancischiJN (2014). Dual effects of Rho-kinase inhibitors on a rat model of inflammatory pain. Pain Res Manag, 19:e172-178.24992453 10.1155/2014/346105PMC4273717

[45] TatsumiS, MabuchiT, KatanoT, MatsumuraS, AbeT, HidakaH, et al. (2005). Involvement of Rho-kinase in inflammatory and neuropathic pain through phosphorylation of myristoylated alanine-rich C-kinase substrate (MARCKS). Neuroscience, 131:491-498.15708490 10.1016/j.neuroscience.2004.10.022

[46] TatsumiE, YamanakaH, KobayashiK, YagiH, SakagamiM, NoguchiK (2015). RhoA/ROCK pathway mediates p38 MAPK activation and morphological changes downstream of P2Y12/13 receptors in spinal microglia in neuropathic pain. Glia, 63:216-228.25130721 10.1002/glia.22745

[47] TakeshitaN, YoshimiE, HatoriC, KumakuraF, SekiN, ShimizuY (2011). Alleviating effects of AS1892802, a Rho kinase inhibitor, on osteoarthritic disorders in rodents. J Pharmacol Sci, 115:481-489.21325780 10.1254/jphs.10319fp

[48] YoshimiE, YamamotoH, FuruichiY, ShimizuY, TakeshitaN (2010). Sustained analgesic effect of the Rho kinase inhibitor AS1892802 in rat models of chronic pain. J Pharmacol Sci, 114:119-122.20710117 10.1254/jphs.10158sc

[49] Lord-FontaineS, YangF, DiepQ, DerghamP, MunzerS, TremblayP, et al. (2008). Local inhibition of Rho signaling by cell-permeable recombinant protein BA-210 prevents secondary damage and promotes functional recovery following acute spinal cord injury. J Neurotrauma, 25:1309-1322.19061375 10.1089/neu.2008.0613

[50] Alvarez-PerezB, HomsJ, Bosch-MolaM, PuigT, ReinaF, VerduE, et al. (2016). Epigallocatechin-3-gallate treatment reduces thermal hyperalgesia after spinal cord injury by down-regulating RhoA expression in mice. Eur J Pain, 20:341-352.25913854 10.1002/ejp.722

[51] LiQ, LiR, ChuX, AnX, ChenM, YuY, et al. (2022). Ubiquitin-specific peptidase 53 promotes chronic constriction injury-induced neuropathic pain through the RhoA/ROCK pathway. Acta Neurobiol Exp (Wars), 82:468-476.36748970 10.55782/ane-2022-045

[52] FuQ, DengY, ZhouB, LeiJ, PengK, FengC (2023). miR-488-3p alleviates neuropathic pain by regulating target gene ROCK1. Acta Neurobiol Exp (Wars), 83:271-279.37874190 10.55782/ane-2023-2432

[53] CuiW, WuH, YuX, SongT, XuX, XuF (2021). The Calcium Channel alpha2delta1 Subunit: Interactional Targets in Primary Sensory Neurons and Role in Neuropathic Pain. Front Cell Neurosci, 15:699731.34658790 10.3389/fncel.2021.699731PMC8514986

[54] CaloL, CinqueC, PataneM, SchillaciD, BattagliaG, MelchiorriD, et al. (2006). Interaction between ephrins/Eph receptors and excitatory amino acid receptors: possible relevance in the regulation of synaptic plasticity and in the pathophysiology of neuronal degeneration. J Neurochem, 98:1-10.16805791 10.1111/j.1471-4159.2006.03844.x

[55] SongXJ, ZhengJH, CaoJL, LiuWT, SongXS, HuangZJ (2008). EphrinB-EphB receptor signaling contributes to neuropathic pain by regulating neural excitability and spinal synaptic plasticity in rats. Pain, 139:168-180.18448254 10.1016/j.pain.2008.03.019

[56] UchidaH, MatsumotoM, UedaH (2009). Profiling of BoNT/C3-reversible gene expression induced by lysophosphatidic acid: ephrinB1 gene up-regulation underlying neuropathic hyperalgesia and allodynia. Neurochem Int, 54:215-221.19111589 10.1016/j.neuint.2008.11.004

[57] RottkampCA, LoburKJ, WladykaCL, LuckyAK, O'GormanS (2008). Pbx3 is required for normal locomotion and dorsal horn development. Dev Biol, 314:23-39.18155191 10.1016/j.ydbio.2007.10.046

[58] KiedrowskiL, CzyzA, BaranauskasG, LiXF, LyttonJ (2004). Differential contribution of plasmalemmal Na/Ca exchange isoforms to sodium-dependent calcium influx and NMDA excitotoxicity in depolarized neurons. J Neurochem, 90:117-128.15198672 10.1111/j.1471-4159.2004.02462.x

[59] DongCR, ZhangWJ, LuoHL (2022). Association between P2X3 receptors and neuropathic pain: As a potential therapeutic target for therapy. Biomed Pharmacother, 150:113029.35489283 10.1016/j.biopha.2022.113029

[60] QiaoWL, LiQ, HaoJW, WeiS, LiXM, LiuTT, et al. (2022). Enhancement of P2X3 Receptor-Mediated Currents by Lysophosphatidic Acid in Rat Primary Sensory Neurons. Front Pharmacol, 13:928647.35795546 10.3389/fphar.2022.928647PMC9251206

[61] Souza Monteiro de AraujoD, NassiniR, GeppettiP, De LoguF (2020). TRPA1 as a therapeutic target for nociceptive pain. Expert Opin Ther Targets, 24:997-1008.32838583 10.1080/14728222.2020.1815191PMC7610834

[62] PaldyE, SimonettiM, WorzfeldT, BaliKK, VicunaL, OffermannsS, et al. (2017). Semaphorin 4C Plexin-B2 signaling in peripheral sensory neurons is pronociceptive in a model of inflammatory pain. Nat Commun, 8:176.28765520 10.1038/s41467-017-00341-wPMC5539317

[63] Shi WG, Yao Y, Liang YJ, Lei J, Feng SY, Zhang ZX, et al. (2024). Activation of TGR5 in the injured nerve site according to a prevention protocol mitigates partial sciatic nerve ligation-induced neuropathic pain by alleviating neuroinflammation. Pain. 10.1097/j.pain.0000000000003460 PMC12067609 39450924

[64] ZhuA, ShenL, XuL, ChenW, HuangY (2018). Wnt5a mediates chronic post-thoracotomy pain by regulating non-canonical pathways, nerve regeneration, and inflammation in rats. Cell Signal, 44:51-61.29339085 10.1016/j.cellsig.2018.01.017

[65] AhnDK, LeeSY, HanSR, JuJS, YangGY, LeeMK, et al. (2009). Intratrigeminal ganglionic injection of LPA causes neuropathic pain-like behavior and demyelination in rats. Pain, 146:114-120.19665300 10.1016/j.pain.2009.07.012

[66] UedaH (2017). Lysophosphatidic acid signaling is the definitive mechanism underlying neuropathic pain. Pain, 158 Suppl 1:S55-S65.28151833 10.1097/j.pain.0000000000000813

[67] InoueM, YamaguchiA, KawakamiM, ChunJ, UedaH (2006). Loss of spinal substance P pain transmission under the condition of LPA1 receptor-mediated neuropathic pain. Mol Pain, 2:25.16914035 10.1186/1744-8069-2-25PMC1562366

[68] UedaH (2008). Peripheral mechanisms of neuropathic pain - involvement of lysophosphatidic acid receptor-mediated demyelination. Mol Pain, 4:11.18377664 10.1186/1744-8069-4-11PMC2365930

[69] SchaksM, GiannoneG, RottnerK (2019). Actin dynamics in cell migration. Essays Biochem, 63:483-495.31551324 10.1042/EBC20190015PMC6823167

[70] WangW, HalaszE, Townes-AndersonE (2019). Actin Dynamics, Regulated by RhoA-LIMK-Cofilin Signaling, Mediates Rod Photoreceptor Axonal Retraction After Retinal Injury. Invest Ophthalmol Vis Sci, 60:2274-2285.31112612 10.1167/iovs.18-26077PMC6530517

[71] ZulaufL, CosteO, MarianC, MoserC, BrenneisC, NiederbergerE (2009). Cofilin phosphorylation is involved in nitric oxide/cGMP-mediated nociception. Biochem Biophys Res Commun, 390:1408-1413.19896457 10.1016/j.bbrc.2009.10.166

[72] BonetIJM, Staurengo-FerrariL, AraldiD, GreenPG, LevineJD (2022). Second messengers mediating high-molecular-weight hyaluronan-induced antihyperalgesia in rats with chemotherapy-induced peripheral neuropathy. Pain, 163:1728-1739.34913881 10.1097/j.pain.0000000000002558PMC9167889

[73] BonetIJM, AraldiD, KhomulaEV, BogenO, GreenPG, LevineJD (2020). Mechanisms Mediating High-Molecular-Weight Hyaluronan-Induced Antihyperalgesia. J Neurosci, 40:6477-6488.32665406 10.1523/JNEUROSCI.0166-20.2020PMC7486649

[74] BonetIJM, KhomulaEV, AraldiD, GreenPG, LevineJD (2021). PI3Kgamma/AKT Signaling in High Molecular Weight Hyaluronan (HMWH)-Induced Anti-Hyperalgesia and Reversal of Nociceptor Sensitization. J Neurosci, 41:8414-8426.34417329 10.1523/JNEUROSCI.1189-21.2021PMC8496198

[75] FerrariLF, KhomulaEV, AraldiD, LevineJD (2018). CD44 Signaling Mediates High Molecular Weight Hyaluronan-Induced Antihyperalgesia. J Neurosci, 38:308-321.29175954 10.1523/JNEUROSCI.2695-17.2017PMC5761612

[76] JiangBC, LiuT, GaoYJ (2020). Chemokines in chronic pain: cellular and molecular mechanisms and therapeutic potential. Pharmacol Ther, 212:107581.32450191 10.1016/j.pharmthera.2020.107581

[77] JiRR, NackleyA, HuhY, TerrandoN, MaixnerW (2018). Neuroinflammation and Central Sensitization in Chronic and Widespread Pain. Anesthesiology, 129:343-366.29462012 10.1097/ALN.0000000000002130PMC6051899

[78] SchwabJM, ConradS, ElbertT, TrautmannK, MeyermannR, SchluesenerHJ (2004). Lesional RhoA+ cell numbers are suppressed by anti-inflammatory, cyclooxygenase-inhibiting treatment following subacute spinal cord injury. Glia, 47:377-386.15293235 10.1002/glia.20031

[79] ZhangX, LiG (2019). P2Y receptors in neuropathic pain. Pharmacol Biochem Behav, 186:172788.31494119 10.1016/j.pbb.2019.172788

[80] LiaoL, QianZY, LiXY, YangDS, LeiBJ, LiHJ, et al. (2021). Disrupting RhoA activity by blocking Arhgef3 expression mitigates microglia-induced neuroinflammation post spinal cord contusion. J Neuroimmunol, 359:577688.34390950 10.1016/j.jneuroim.2021.577688

[81] LiT, LiuT, ChenX, LiL, FengM, ZhangY, et al. (2020). Microglia induce the transformation of A1/A2 reactive astrocytes via the CXCR7/PI3K/Akt pathway in chronic post-surgical pain. J Neuroinflammation, 17:211.32665021 10.1186/s12974-020-01891-5PMC7362409

[82] ZhangY, WangX, JiangC, ChenZ, NiS, FanH, et al. (2022). Rho Kinase Inhibitor Y27632 Improves Recovery After Spinal Cord Injury by Shifting Astrocyte Phenotype and Morphology via the ROCK/NF-kappaB/C3 Pathway. Neurochem Res, 47:3733-3744.36103106 10.1007/s11064-022-03756-0PMC9718714

[83] FanX, ChuR, JiangX, MaP, ChuY, HuaT, et al. (2024). LPAR6 Participates in Neuropathic Pain by Mediating Astrocyte Cells via ROCK2/NF-kappaB Signal Pathway. Mol Neurobiol, 61:8402-8413.38509397 10.1007/s12035-024-04108-5

[84] MaJ, WangZ, ChenS, SunW, GuQ, LiD, et al. (2021). EphA1 Activation Induces Neuropathological Changes in a Mouse Model of Parkinson's Disease Through the CXCL12/CXCR4 Signaling Pathway. Mol Neurobiol, 58:913-925.33057926 10.1007/s12035-020-02122-x

[85] McGaraughtyS, ChuKL, XuJ (2018). Characterization and pharmacological modulation of noci-responsive deep dorsal horn neurons across diverse rat models of pathological pain. J Neurophysiol, 120:1893-1905.30067136 10.1152/jn.00325.2018

[86] VelazquezKT, MohammadH, SweitzerSM (2007). Protein kinase C in pain: involvement of multiple isoforms. Pharmacol Res, 55:578-589.17548207 10.1016/j.phrs.2007.04.006PMC2140050

[87] ArtolaA, VoisinD, DallelR (2020). PKCgamma interneurons, a gateway to pathological pain in the dorsal horn. J Neural Transm (Vienna), 127:527-540.32108249 10.1007/s00702-020-02162-6

[88] Merighi A (2024). Brain-Derived Neurotrophic Factor, Nociception, and Pain. Biomolecules, 14. 10.3390/biom14050539 PMC11118093 38785946

[89] Luo X, Tai WL, Sun L, Pan Z, Xia Z, Chung SK, et al. (2016). Crosstalk between astrocytic CXCL12 and microglial CXCR4 contributes to the development of neuropathic pain. Mol Pain, 12. 10.1177/1744806916636385 PMC4956184 27030717

[90] BaiL, WangX, LiZ, KongC, ZhaoY, QianJL, et al. (2016). Upregulation of Chemokine CXCL12 in the Dorsal Root Ganglia and Spinal Cord Contributes to the Development and Maintenance of Neuropathic Pain Following Spared Nerve Injury in Rats. Neurosci Bull, 32:27-40.26781879 10.1007/s12264-015-0007-4PMC5563752

[91] HoffmanEM, ZhangZ, SchechterR, MillerKE (2016). Glutaminase Increases in Rat Dorsal Root Ganglion Neurons after Unilateral Adjuvant-Induced Hind Paw Inflammation. Biomolecules, 6:10.26771651 10.3390/biom6010010PMC4808804

[92] BlissTV, CollingridgeGL, KaangBK, ZhuoM (2016). Synaptic plasticity in the anterior cingulate cortex in acute and chronic pain. Nat Rev Neurosci, 17:485-496.27307118 10.1038/nrn.2016.68

[93] ChakrabartyA, BlacklockA, SvojanovskyS, SmithPG (2008). Estrogen elicits dorsal root ganglion axon sprouting via a renin-angiotensin system. Endocrinology, 149:3452-3460.18388195 10.1210/en.2008-0061PMC2453086

[94] ChenH, FiresteinBL (2007). RhoA regulates dendrite branching in hippocampal neurons by decreasing cypin protein levels. J Neurosci, 27:8378-8386.17670984 10.1523/JNEUROSCI.0872-07.2007PMC6673065

[95] DillJ, PatelAR, YangXL, BachooR, PowellCM, LiS (2010). A molecular mechanism for ibuprofen-mediated RhoA inhibition in neurons. J Neurosci, 30:963-972.20089905 10.1523/JNEUROSCI.5045-09.2010PMC3904382

[96] BroseK, Tessier-LavigneM (2000). Slit proteins: key regulators of axon guidance, axonal branching, and cell migration. Curr Opin Neurobiol, 10:95-102.10679444 10.1016/s0959-4388(99)00066-5

[97] SasakiY (2003). New aspects of neurotransmitter release and exocytosis: Rho-kinase-dependent myristoylated alanine-rich C-kinase substrate phosphorylation and regulation of neurofilament structure in neuronal cells. J Pharmacol Sci, 93:35-40.14501149 10.1254/jphs.93.35

[98] OhsawaM, MutohJ, YamamotoS, HisaH (2014). Involvement of protein isoprenylation in neuropathic pain induced by sciatic nerve injury in mice. Neurosci Lett, 564:27-31.24486886 10.1016/j.neulet.2014.01.039

[99] SchmidtkoA, TegederI, GeisslingerG (2009). No NO, no pain? The role of nitric oxide and cGMP in spinal pain processing. Trends Neurosci, 32:339-346.19414201 10.1016/j.tins.2009.01.010

[100] CuryY, PicoloG, GutierrezVP, FerreiraSH (2011). Pain and analgesia: The dual effect of nitric oxide in the nociceptive system. Nitric Oxide, 25:243-254.21723953 10.1016/j.niox.2011.06.004

[101] SousaAM, PradoWA (2001). The dual effect of a nitric oxide donor in nociception. Brain Res, 897:9-19.11282353 10.1016/s0006-8993(01)01995-3

[102] Li ZZ, Han WJ, Sun ZC, Chen Y, Sun JY, Cai GH, et al. (2021). Extracellular matrix protein laminin beta1 regulates pain sensitivity and anxiodepression-like behaviors in mice. J Clin Invest, 131. 10.1172/JCI146323 PMC8321574 34156983

[103] HanleyJG (2014). Actin-dependent mechanisms in AMPA receptor trafficking. Front Cell Neurosci, 8:381.25429259 10.3389/fncel.2014.00381PMC4228833

[104] ColeJC, VillaBR, WilkinsonRS (2000). Disruption of actin impedes transmitter release in snake motor terminals. J Physiol, 525 Pt 3:579-586.10856113 10.1111/j.1469-7793.2000.t01-2-00579.xPMC2269967

[105] SankaranarayananS, AtluriPP, RyanTA (2003). Actin has a molecular scaffolding, not propulsive, role in presynaptic function. Nat Neurosci, 6:127-135.12536209 10.1038/nn1002

[106] KwonM, AltinM, DuenasH, AlevL (2014). The role of descending inhibitory pathways on chronic pain modulation and clinical implications. Pain Pract, 14:656-667.24256177 10.1111/papr.12145

[107] BenarrochEE (2008). Descending monoaminergic pain modulation: bidirectional control and clinical relevance. Neurology, 71:217-221.18625968 10.1212/01.wnl.0000318225.51122.63

[108] BravoL, Llorca-TorralbaM, BerrocosoE, MicoJA (2019). Monoamines as Drug Targets in Chronic Pain: Focusing on Neuropathic Pain. Front Neurosci, 13:1268.31942167 10.3389/fnins.2019.01268PMC6951279

[109] DuffyP, SchmandkeA, SchmandkeA, SigworthJ, NarumiyaS, CaffertyWB, et al. (2009). Rho-associated kinase II (ROCKII) limits axonal growth after trauma within the adult mouse spinal cord. J Neurosci, 29:15266-15276.19955379 10.1523/JNEUROSCI.4650-09.2009PMC2855556

[110] MuellerBK, MackH, TeuschN (2005). Rho kinase, a promising drug target for neurological disorders. Nat Rev Drug Discov, 4:387-398.15864268 10.1038/nrd1719

[111] StrattonHJ, KhannaR (2020). Sculpting Dendritic Spines during Initiation and Maintenance of Neuropathic Pain. J Neurosci, 40:7578-7589.32998955 10.1523/JNEUROSCI.1664-20.2020PMC7531544

[112] SchillY, BijataM, KopachO, CherkasV, Abdel-GalilD, BohmK, et al. (2020). Serotonin 5-HT(4) receptor boosts functional maturation of dendritic spines via RhoA-dependent control of F-actin. Commun Biol, 3:76.32060357 10.1038/s42003-020-0791-xPMC7021812

[113] ZhuZ, LuJ, WangS, PengW, YangY, ChenC, et al. (2022). Acrolein, an endogenous aldehyde induces synaptic dysfunction in vitro and in vivo: Involvement of RhoA/ROCK2 pathway. Aging Cell, 21:e13587.35315217 10.1111/acel.13587PMC9009232

[114] LarsonCM, WilcoxGL, FairbanksCA (2019). The Study of Pain in Rats and Mice. Comp Med, 69:555-570.31822322 10.30802/AALAS-CM-19-000062PMC6935695

[115] ZhangLQ, ZhangW, LiT, YangT, YuanX, ZhouY, et al. (2021). GLP-1R activation ameliorated novel-object recognition memory dysfunction via regulating hippocampal AMPK/NF-kappaB pathway in neuropathic pain mice. Neurobiol Learn Mem, 182:107463.34015440 10.1016/j.nlm.2021.107463

[116] GeMM, ChenSP, ZhouYQ, LiZ, TianXB, GaoF, et al. (2019). The therapeutic potential of GABA in neuron-glia interactions of cancer-induced bone pain. Eur J Pharmacol, 858:172475.31228456 10.1016/j.ejphar.2019.172475

[117] MogilJS (2019). The translatability of pain across species. Philos Trans R Soc Lond B Biol Sci, 374:20190286.31544615 10.1098/rstb.2019.0286PMC6790385

[118] SadlerKE, MogilJS, StuckyCL (2022). Innovations and advances in modelling and measuring pain in animals. Nat Rev Neurosci, 23:70-85.34837072 10.1038/s41583-021-00536-7PMC9098196

[119] LoirandG (2015). Rho Kinases in Health and Disease: From Basic Science to Translational Research. Pharmacol Rev, 67:1074-1095.26419448 10.1124/pr.115.010595

[120] ChatterjeeS, PatraD, GhoshP, BanerjeeS, ChowdhuryKD, ChakrabortyP, et al. (2022). Activity of ROCKII not ROCKI promotes pulmonary metastasis of melanoma cells via modulating Smad2/3-MMP9 and FAK-Src-VEGF signalling. Cell Signal, 97:110389.35718242 10.1016/j.cellsig.2022.110389

[121] MaruhashiT, HigashiY (2021). An overview of pharmacotherapy for cerebral vasospasm and delayed cerebral ischemia after subarachnoid hemorrhage. Expert Opin Pharmacother, 22:1601-1614.33823726 10.1080/14656566.2021.1912013

[122] FracassiA, MarangoniM, RossoP, PallottiniV, FioramontiM, SiteniS, et al. (2019). Statins and the Brain: More than Lipid Lowering Agents? Curr Neuropharmacol, 17:59-83.28676012 10.2174/1570159X15666170703101816PMC6341496

[123] KobayashiK, TakahashiM, MatsushitaN, MiyazakiJ, KoikeM, YaginumaH, et al. (2004). Survival of developing motor neurons mediated by Rho GTPase signaling pathway through Rho-kinase. J Neurosci, 24:3480-3488.15071095 10.1523/JNEUROSCI.0295-04.2004PMC6729735

[124] KobayashiK, SanoH, KatoS, KurodaK, NakamutaS, IsaT, et al. (2016). Survival of corticostriatal neurons by Rho/Rho-kinase signaling pathway. Neurosci Lett, 630:45-52.27424794 10.1016/j.neulet.2016.07.020

[125] KochJC, LehaA, BidnerH, CordtsI, DorstJ, GuntherR, et al. (2024). Safety, tolerability, and efficacy of fasudil in amyotrophic lateral sclerosis (ROCK-ALS): a phase 2, randomised, double-blind, placebo-controlled trial. Lancet Neurol, 23:1133-1146.39424560 10.1016/S1474-4422(24)00373-9PMC12741558

[126] WangW, HuangW, LiuJ, ZhangZ, JiR, WuC, et al. (2023). Electric field promotes dermal fibroblast transdifferentiation through activation of RhoA/ROCK1 pathway. Int J Med Sci, 20:1326-1335.37786441 10.7150/ijms.86215PMC10542021

[127] WolffAW, PeineJ, HoflerJ, ZurekG, HemkerC, LingorP (2024). SAFE-ROCK: A Phase I Trial of an Oral Application of the ROCK Inhibitor Fasudil to Assess Bioavailability, Safety, and Tolerability in Healthy Participants. CNS Drugs, 38:291-302.38416402 10.1007/s40263-024-01070-7PMC10980656

[128] GutekunstCA, TungJK, McDougalME, GrossRE (2016). C3 transferase gene therapy for continuous conditional RhoA inhibition. Neuroscience, 339:308-318.27746349 10.1016/j.neuroscience.2016.10.022PMC5118153

[129] TanJ, LiuG, ZhuX, WuZ, WangN, ZhouL, et al. (2019). Lentiviral Vector-Mediated Expression of Exoenzyme C3 Transferase Lowers Intraocular Pressure in Monkeys. Mol Ther, 27:1327-1338.31129118 10.1016/j.ymthe.2019.04.021PMC6612778

